# Catalytic Subunit 1 of Protein Phosphatase 2A Is a Subunit of the STRIPAK Complex and Governs Fungal Sexual Development

**DOI:** 10.1128/mBio.00870-16

**Published:** 2016-06-21

**Authors:** Anna Beier, Ines Teichert, Christoph Krisp, Dirk A. Wolters, Ulrich Kück

**Affiliations:** a Lehrstuhl für Allgemeine und Molekulare Botanik, Ruhr-Universität, Bochum, Germany; b Lehrstuhl für Analytische Chemie, Ruhr-Universität, Bochum, Germany

## Abstract

The generation of complex three-dimensional structures is a key developmental step for most eukaryotic organisms. The details of the molecular machinery controlling this step remain to be determined. An excellent model system to study this general process is the generation of three-dimensional fruiting bodies in filamentous fungi like *Sordaria macrospora*. Fruiting body development is controlled by subunits of the highly conserved striatin-interacting phosphatase and kinase (STRIPAK) complex, which has been described in organisms ranging from yeasts to humans. The highly conserved heterotrimeric protein phosphatase PP2A is a subunit of STRIPAK. Here, catalytic subunit 1 of PP2A was functionally characterized. The Δ*pp2Ac1* strain is sterile, unable to undergo hyphal fusion, and devoid of ascogonial septation. Further, PP2Ac1, together with STRIPAK subunit PRO22, governs vegetative and stress-related growth. We revealed *in vitro* catalytic activity of wild-type PP2Ac1, and our *in vivo* analysis showed that inactive PP2Ac1 blocks the complementation of the sterile deletion strain. Tandem affinity purification, followed by mass spectrometry and yeast two-hybrid analysis, verified that PP2Ac1 is a subunit of STRIPAK. Further, these data indicate links between the STRIPAK complex and other developmental signaling pathways, implying the presence of a large interconnected signaling network that controls eukaryotic developmental processes. The insights gained in our study can be transferred to higher eukaryotes and will be important for understanding eukaryotic cellular development in general.

## IMPORTANCE

The striatin-interacting phosphatase and kinase (STRIPAK) complex is highly conserved from yeasts to humans and is an important regulator of numerous eukaryotic developmental processes, such as cellular signaling and cell development. Although functional insights into the STRIPAK complex are accumulating, the detailed molecular mechanisms of single subunits are only partially understood. The first fungal STRIPAK was described in *Sordaria macrospora*, which is a well-established model organism used to study the formation of fungal fruiting bodies, three-dimensional organ-like structures. We analyzed STRIPAK subunit PP2Ac1, catalytic subunit 1 of protein phosphatase PP2A, to study the importance of the catalytic activity of this protein during sexual development. The results of our yeast two-hybrid analysis and tandem affinity purification, followed by mass spectrometry, indicate that PP2Ac1 activity connects STRIPAK with other signaling pathways and thus forms a large interconnected signaling network.

## INTRODUCTION

Reversible protein phosphorylations are among the most common posttranslational modifications (1) that are involved in various signaling pathways controlling a multitude of different processes, such as cell proliferation and sexual propagation (2, 3). Kinases and phosphatases regulate protein phosphorylation mostly on serine (S) and threonine (T) residues and less frequently on tyrosine (Y) residues. In mammals, up to 400 kinases exist, while lower eukaryotes, such as filamentous fungi, possess about 100 kinases (4, 5). Kinases are classified into families based on substrate specificity and domain structure (6).

The number of phosphatases is similar to the number of kinases. However, significantly fewer phosphatase genes than kinase genes exist, since phosphatase diversity is based on the multimeric nature of most protein phosphatases. For example, major and highly abundant S/T protein phosphatase 2A (PP2A) is a heterotrimeric protein phosphatase. A scaffolding subunit (PP2AA) and a catalytic subunit (PP2Ac) form a core complex, which is joined by a B-type regulatory subunit to complete the functional holoenzyme. The PP2A core complex is well conserved, and mammalian cells contain two isoforms each of PP2AA and PP2Ac (7). In fungi, PP2AA is encoded by a single gene, while two genes exist for PP2Ac isoforms that control developmental processes such as vegetative growth, hyphal fusion, virulence, and asexual and sexual propagation (8–11).

Regulatory PP2A subunits mediate substrate specificity and are classified into four families, called B, B′, B′′, and B′′′. Although members of these protein families within a single species show rather low sequence similarity, homologs of a given family are well conserved from yeasts to humans. The B′′′ subunits are the latest members of the regulatory subunits and are referred to as striatins (12). Striatins were initially detected in structures of the central nervous system, mostly in the striatum, and were the denominating subunits of the striatin-interacting phosphatase and kinase (STRIPAK) complex (13–15). Besides striatin, further subunits of STRIPAK are PP2AA and PP2Ac, striatin-interacting protein 1/2 (STRIP1/2), sarcolemmal membrane-associated protein (SLMAP), monopolar spindle-one-binder homolog 3 (Mob3), cerebral cavernous malformation 3 protein, and germinal-center-like kinases (15). STRIPAK coordinates key cellular processes such as cytoskeleton organization, cell migration, and cell size, as well as morphology control, in mammalians (15). In fungi, this complex regulates sexual and asexual development, hyphal fusion, and virulence (14, 15). Although the functions of STRIPAK complexes have been discovered in recent years, the molecular function of distinct subunits remains largely obscure.

We previously characterized the STRIPAK complex of the filamentous fungus *Sordaria macrospora*, which comprises, besides PP2AA, the striatin homolog PRO11, the STRIP1/2 homolog PRO22, the SLMAP homolog PRO45, the Mob3 homolog SmMOB3, and the germinal-center kinases SmKIN3 and SmKIN24 (14, 16). Previous evidence from protein-protein interaction studies indicated that catalytic subunit 1 of PP2A (PP2Ac1) is also part of fungal STRIPAK (17, 18). Therefore, the aim of this study was to functionally characterize PP2Ac1 in the filamentous fungus *S. macrospora*. We show that active PP2Ac1 is required for fruiting body formation, hyphal fusion, and vegetative growth. Genetic studies support the idea of a core STRIPAK complex comprising PP2A and the STRIP1/2 homolog PRO22, which plays a role in the septation of early reproductive structures. Finally, yeast two-hybrid (Y2H) analysis and tandem affinity purification (TAP)-mass spectrometry (MS) with PP2Ac1 as bait revealed that PP2Ac1 directly interacts with PRO22 and PP2AA. Our studies indicate that catalytically active PP2Ac1 is a major regulator of fungal sexual development and must be considered a key component of the fungal STRIPAK complex.

## RESULTS

### The *pp2Ac1* gene governs fungal sexual development and hyphal fusion.

Previous TAP-MS data obtained with PRO22 as bait supported the idea that PP2Ac1 is part of the fungal STRIPAK complex (17) and stimulated our interest in its functional characterization. Annotation of the *S. macrospora* genome identified two genes encoding catalytic subunits 1 (PP2Ac1, SMAC_04678) and 2 (PP2Ac2, SMAC_04756) of PP2A. Both are highly homologous to their human homologs (see Fig. S1 in the supplemental material). We constructed *pp2Ac1* and *pp2Ac2* deletion mutants. We failed to generate a Δ*pp2Ac2* mutant, since the deletion is most probably lethal, as in other fungi (19–21). However, we successfully generated a Δ*pp2Ac1* strain, where *pp2Ac1* is replaced with a hygromycin B resistance cassette. The deletion was confirmed by PCR analysis and Southern hybridization (see Fig. S2A to C in the supplemental material).

**FIG 1:**
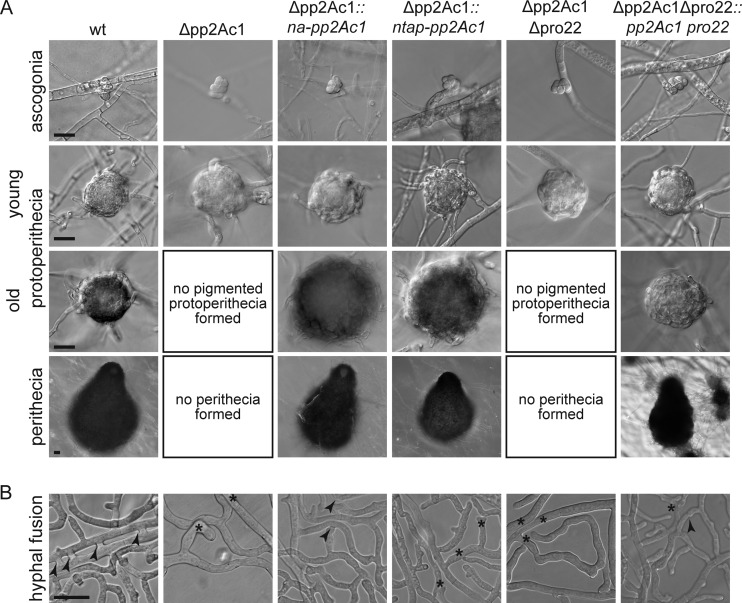
Phenotypic characterization of Δ*pp2Ac1* and Δ*pp2Ac1* Δ*pro22* mutant strains. (A) Microscopic documentation of sexual development of strains grown on BMM-coated slides incubated for 2 to 7 days. wt, wild type. (B) Hyphal fusion was analyzed on cellophane-covered solid MMS 1 to 3 days postinoculation in a region 5 to 10 mm off the colony edges. Arrowheads and asterisks indicate hyphal fusion events and hyphae making contact but lacking fusion bridges, respectively. All scale bars, 20 µm.

We analyzed the sexual development of the Δ*pp2Ac1* mutant strain in comparison to that of the wild type. The life cycle of *S. macrospora* is completed within 7 days. Starting from germinating sexual spores (ascospores), the fungus forms vegetative mycelium. Some hyphae differentiate into female gametangia, the ascogonia, which develop into immature fruiting bodies (protoperithecia) and then into pear-shaped fruiting bodies called perithecia. They protect the sexual sporangia (asci), each containing eight ascospores (22). We observed that after growth for 2 to 7 days on BMM (biomalt and maize extract medium)-coated slides, the Δ*pp2Ac1* strain generates small protoperithecia but never pigmented protoperithecia or perithecia (Fig. 1A). This phenotype of the Δ*pp2Ac1* mutant strain resembles the previously described pro phenotype, which has also been described in strains lacking genes for STRIPAK subunits (14). When we transformed the deletion strain with the wild-type *pp2Ac1* gene controlled by its own promoter, we observed restoration of sexual development (Fig. 1A). Fertility was also regained after the transformation of a recombinant gene encoding an N-terminal fusion of the TAP tag to PP2Ac1 (*ntap-pp2Ac1*) (Fig. 1A). The number of perithecia in the NTAP-PP2Ac1 strain is lower than that in the wild type (see Fig. S2D in the supplemental material). However, strains remained sterile when we used a construct for C-terminally tagged PP2Ac1 (data not shown).

We and others observed previously that STRIPAK mutants have defects in both sexual development and hyphal fusion (14). Thus, we analyzed hyphal fusion in vegetative mycelium of the wild-type and Δ*pp2Ac1* mutant strains. Unlike the wild type, which frequently formed hyphal fusion bridges, the deletion strain was unable to undergo hyphal fusion (Fig. 1B). Reintroduction of the wild-type gene with its own promoter into the Δ*pp2Ac1* strain rescued the mutant phenotype. However, we were unable to show hyphal fusion in the Δ*pp2Ac1* strain containing overexpressed *ntap-pp2Ac1*.

### PP2Ac1 is required for ascogonial septation.

**FIG 2:**
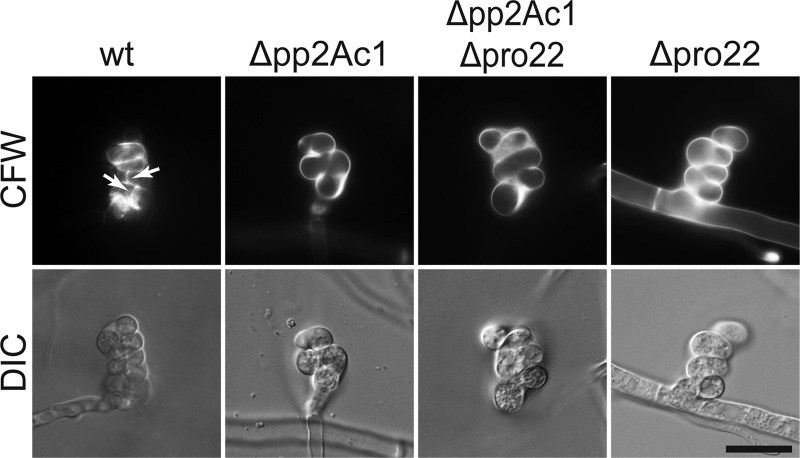
Ascogonial septation depends on PP2Ac1 and PRO22. Septum formation in ascogonial coils was documented in strains grown on BMM-coated slides for 4 days. Cell walls were stained with CFW. Arrows indicate septa in the ascogonial coils (only in the wild type [wt]). Scale bar, 20 µm.

Previously, aseptate ascogonia were described as being a unique feature of mutant pro22 (23). Here, we observed the same phenotype in the Δ*pp2Ac1* deletion strain (Fig. 2). This observation indicates that PRO22 and PP2Ac1 have a mutual function in the same signaling pathway that is distinct from STRIPAK signaling. To test this hypothesis, crosses were performed to generate a Δ*pp2Ac1* Δ*pro22* double deletion strain (see Fig. S3A in the supplemental material). The corresponding homokaryotic isolates were defective in sexual development and hyphal fusion, and the wild-type phenotype was restored by transformation of the double deletion strain with both wild-type genes (Fig. 1A and B). To quantify septum formation in ascogonial coils of the Δ*pp2Ac1*, Δ*pro22*, and Δ*pp2Ac1* Δ*pro22* strains compared to that in the wild type (Fig. 2), ascogonial septa were visualized by staining the cell wall with Calcofluor White M2R (CFW; Sigma Aldrich, St. Louis, MO, United States) (23). Screening of 200 ascogonia of each strain showed that intercalary septa occurred only in the wild type (Fig. 2), where all ascogonia showed septation.

### Vegetative and stress-related growth is governed by STRIPAK subunits.

**FIG 3:**
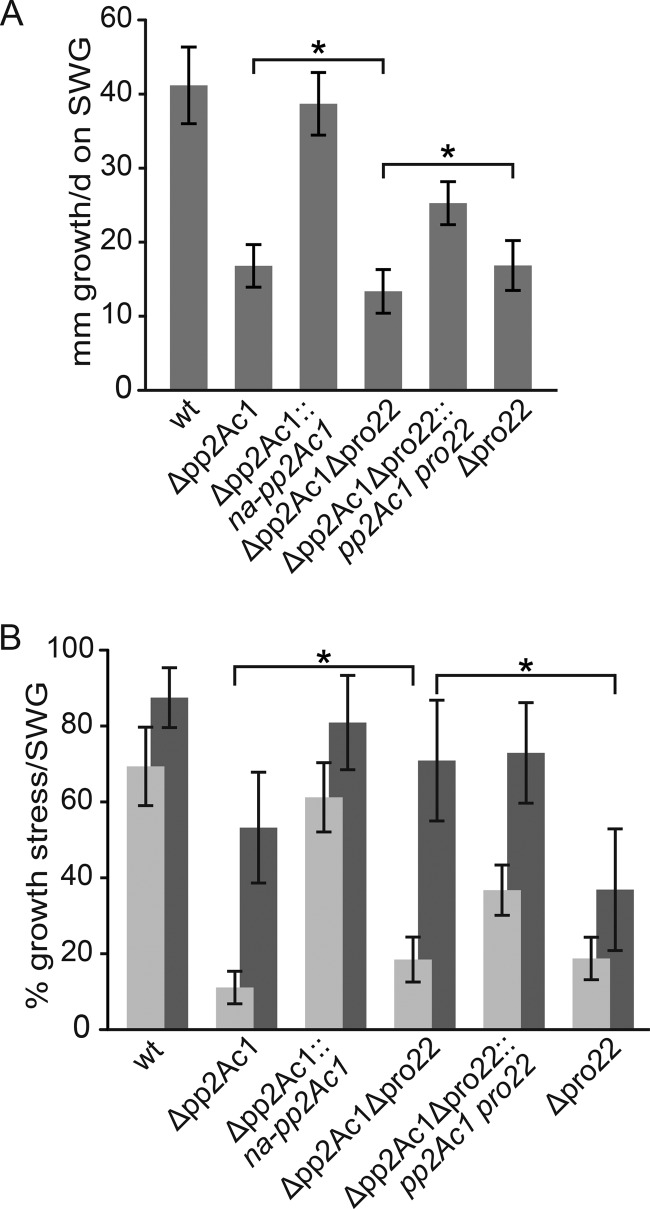
Vegetative and stress-related growth defects of Δ*pp2Ac1* and Δ*pp2Ac1* Δ*pro22* mutant strains. (A) Vegetative growth on SWG medium was investigated in race tubes for 6 days, and growth fronts were marked every 24 h. (B) Cell wall stress- and osmotic stress-related growth was tested on SWG medium containing 250 µg/ml CFW (dark gray) or 0.6 M glucose (light gray). All error bars indicate standard deviations from three independent experiments with three replicates each. All brackets indicate significant differences in growth according to Student’s *t* test with *P* < 0.01 (*) for single and double deletion strains. wt, wild type.

Mutations in many developmental genes have been linked to delayed vegetative and stress-related growth (24, 25). We compared the vegetative growth rates of the wild-type and Δ*pp2Ac1*, Δ*pro22*, and Δ*pp2Ac1* Δ*pro22* mutant strains. While the wild type grows approximately 41 mm/day, both single deletion strains exhibited a significantly reduced growth rate of about 17 mm/day. This reduction was even more severe in the double deletion strain, which grew only 13.4 mm/day (Fig. 3A). We tested different growth conditions to trigger endoplasmic reticulum (ER), oxidative, cell wall, and osmotic stresses. Only osmotic stress, induced by high sugar concentrations, and cell wall stress caused a significant growth defect in the deletion strains (Fig. 3B), while all of the other conditions tested revealed no defect (see Fig. S3C in the supplemental material). In comparison to the Δ*pro22* and Δ*pp2Ac1* mutant strains, the double deletion strain exhibited a similar sensitivity to osmotic stress, while cell wall stress-related growth was slightly restored in the Δ*pp2Ac1* Δ*pro22* strain (Fig. 3B). Thus, the double deletion strain partially bypasses the cell wall stress-sensitive phenotype of the single deletion strains. Once again, the mutants regained wild-type-like growth rates after transformation with the corresponding wild-type genes (Fig. 3A and B).

### Defining the interaction network of PP2Ac1.

Previously, we described the STRIPAK complex of *S. macrospora* by TAP-MS with the STRIP1/2 homolog PRO22 as bait (17). This approach identified PP2Ac1 as a putative STRIPAK subunit. Here, we studied the interaction network of PP2Ac1 by using TAP-MS in a similar approach. For this purpose, we constructed the Δ*pp2Ac1*::*ntap-pp2Ac1* strain, which is fertile and thus confirms the functionality of the *ntap-pp2Ac1* fusion gene in this developmental process (Fig. 1A). We identified more than 350 proteins in four TAP-MS experiments (see Dataset S1 in the supplemental material). We found 315 proteins in total and obtained putative interaction partners of PP2Ac1 by subtracting the unspecific background from several TAP-MS and affinity purification (AP)-MS experiments (25) and eliminating ribosomal proteins. Eight proteins were found in at least three experiments with a minimum mean of five spectral counts (Table 1). The protein identified with the highest number of spectral counts, besides PP2Ac1, was PRO22 (SMAC_02580). In all of our experiments, we found two regulators of PP2Ac1 in high abundance, called two A phosphatase-associated protein 42 (TAP42; SMAC_00948) and protein phosphatase two A phosphatase activator 1 (PTPA1; SMAC_03446). Furthermore, we identified a protein probably involved in RNA polymerase II degradation (SMAC_00817), a putative poly(A) RNA binding protein (SMAC_02147), and two proteins of unknown function (SMAC_00681, SMAC_08770).

**TABLE 1:** Putative interaction partners of PP2Ac1 identified by TAP-MS

*S. macrospora* identifier	Protein	Function	Spectral count (peptide count)				Ø MS1-4 PSMs^a^
			MS1	MS2	MS3	MS4	
SMAC_04678	PP2Ac1	Catalytic subunit of PP2A	34 (7)	55 (7)	325 (16)	125 (6)	134.75
SMAC_02580	PRO22	Developmental protein	84 (25)	51 (20)	174 (45)	6 (6)	78.75
SMAC_03446	PTPA1	PP2A activator	32 (16)	14 (12)	75 (23)	9 (9)	32.5
SMAC_00948	TAP42	Associated with PP2A	28 (9)	20 (8)	36 (14)	4 (3)	22
SMAC_00817	DEF	RNA polymerase II degradation	14 (7)	7 (6)	17 (10)	8 (5)	11.5
SMAC_02147	PUB1	Poly(A) RNA binding	7 (4)	3 (2)	8 (4)	2 (2)	5
SMAC_00681	Hypothetical		4 (4)	4 (4)	17 (12)		6.25
SMAC_08770	Hypothetical		4 (4)	2 (2)	16 (11)		5.5

a MS1 to MS4, mass spectrometry runs 1 to 4, respectively; Ø, average value; PSMs, peptide spectral matches (spectral counts).

We analyzed our TAP-MS data for signaling proteins that did not fit the above-mentioned criteria. The PP2A scaffold protein PP2AA (SMAC_01919) and the B-type regulatory subunit of PP2A, CDC55 (SMAC_04241) were identified in two experiments and one experiment, respectively. Furthermore, we detected the scaffold protein for the cell wall integrity (CWI) mitogen-activated protein kinase (MAPK) pathway, PRO40 (SMAC_04815), and the CWI pathway components RHO1 (SMAC_06239) (25) and GTPase-activating protein SAC7 (SMAC_02232), which is also a target of the target of rapamycin complex (TORC) (26). Moreover, we discovered protein kinase GSK-3 (SMAC_04408), which we identified previously in TAP-MS with PRO45 as bait (27). In addition, three putative methyltransferases (SMAC_09155, SMAC_06301, and SMAC_09840) that might be involved in posttranslational modification of PP2Ac1 were detected.

### Y2H analysis indicates an interaction between STRIPAK and the CWI pathway.

**FIG 4:**
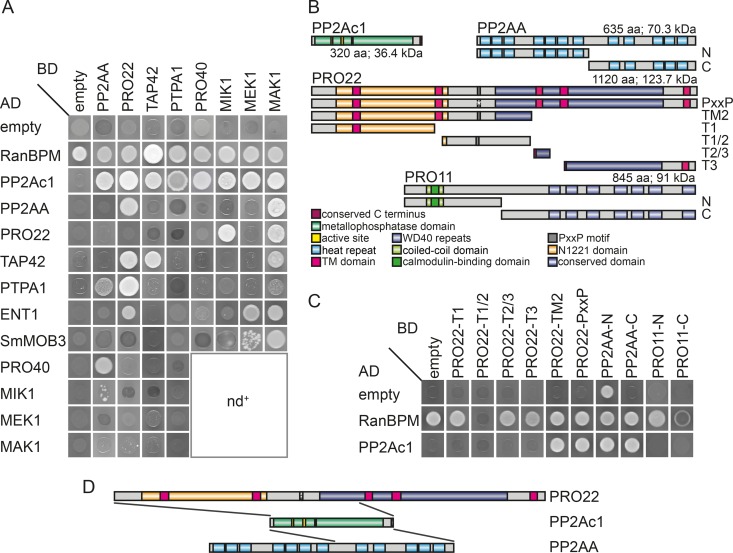
Interaction of STRIPAK components and CWI pathway module subunits. (A) Interaction of fusion proteins was tested in a Y2H analysis by measuring the growth of diploid strains on synthetic defined (SD) medium lacking adenine, histidine, leucine, and tryptophan. The corresponding growth control is shown in Fig. S4A in the supplemental material. nd^+^, not determined in this study. (B) Protein domain structure of PP2Ac1, PP2AA, PRO22, and PRO11. The fragments used for interaction site analysis are depicted below the full-length proteins. TM, transmembrane; aa, amino acids. (C) Interaction of PRO22, PP2AA, and PRO11 subfragments with PP2Ac1 was tested on SD medium lacking adenine, histidine, leucine, and tryptophan/uracil, and the corresponding growth control is shown in Fig. S4B in the supplemental material. (D) Schematic illustration of interaction sites of PP2Ac1 and PRO22 and of PP2Ac1 and PP2AA. Gray lines indicate the interaction sites determined in panel C.

Our TAP-MS data gave us an insight into the composition of the protein complex containing PP2Ac1. However, direct interactions within this complex remained elusive. The TAP-MS analysis presented here, together with previous TAP- and AP-MS analyses, emphasizes a putative connection between STRIPAK and the CWI pathway (17, 25). Similar to STRIPAK, the CWI pathway is also involved in sexual development, hyphal fusion, and vegetative, as well as stress-related, growth (25). Thus, we performed Y2H studies to investigate direct physical interactions between known STRIPAK subunits, putative regulators of PP2Ac1, and the MAPK kinase kinase MIK1, the MAPK kinase MEK1, and the MAPK MAK1, as well as the scaffold protein PRO40 of the CWI pathway. We analyzed the putative interaction partners PP2Ac1, TAP42, PTPA1, PRO22, PP2AA, SmMOB3, MIK1, MEK1, MAK1, PRO40, and epsin ENT1. Epsins in yeast interact with STRIPAK subunits and putatively link this signaling complex to endocytotic events (26). Mating of yeast strains PJ69-4a and PJ69-4α carrying Gal4 activation domain (AD) or DNA-binding domain (BD) fusions of the above-mentioned proteins resulted in diploid strains that were tested for growth (see Fig. S4 in the supplemental material) and reporter gene activity (Fig. 4A). PP2Ac1 physically interacts with STRIPAK subunits PP2AA and PRO22 and with its regulators PTPA1 and TAP42, with the latter forming a homodimer (Fig. 4A). PP2Ac1 showed interactions with all tested components of the CWI pathway. In addition, we identified several other direct interactions between STRIPAK and CWI pathway subunits (Fig. 4A), which emphasizes a link between both protein complexes. In detail, we confirmed putative protein-protein interactions between PRO22 and MAK1 and between PRO40 and PP2AA (Fig. 4A) that were identified by previous TAP- and AP-MS approaches (17, 25). Furthermore, MAK1 exhibited an interaction with TAP42 and ENT1 that was not described previously.

Since BD-PP2Ac1 and BD-PRO11 fusion proteins show transactivation, we tested the binding of PP2Ac1 to subfragments of PRO11, which is the homolog of mammalian striatin and thus the regulatory subunit of PP2A in STRIPAK. To determine the interaction sites of PRO22 and PP2AA that mediate binding to PP2Ac1, we used subfragments as displayed in Fig. 4B. Growth tests for reporter gene activity were performed with diploid yeast strains carrying fragments as indicated in Fig. 4C. We deduced that PP2Ac1 interacts with PRO22-PxxP and PRO22-TM2 but not with PRO22-T1, PRO22-T2/3, and PRO22-T3. PRO22-PxxP is full-length PRO22 with a mutation in the PxxP motif, which is supposed to mediate protein-protein interaction in large protein complexes (28), while PRO22-TM2 contains the N1221 domain, the PxxP domain, and a fragment of the conserved domain. In summary, the N1221 domain is involved in the interaction between PRO22 and PP2Ac1, while the conserved domain and the PxxP motif are not essential for this interaction (Fig. 4D). PP2Ac1 and PRO11 subfragments did not show a physical interaction, while PP2Ac1 and PP2AA-C, containing HEAT (huntingtin, elongation factor 3, PP2A, and *Saccharomyces cerevisiae* TOR1) repeats 8 to 12, interacted in the Y2H analysis.

We identified the CWI pathway components as interaction partners of STRIPAK subunits PP2Ac1 and PRO22. Thus, we analyzed the phosphorylation status of MAK1 in both deletion strains and the double deletion strain with an appropriate antibody. As shown in Fig. S5 in the supplemental material, the phosphorylation level of MAK1 is unchanged in the Δ*pp2Ac1* strain and slightly but not significantly higher in the Δ*pro22* and Δ*pp2Ac1* Δ*pro22* strains.

### Catalytically active PP2Ac1 is required for sexual development.

**FIG 5:**
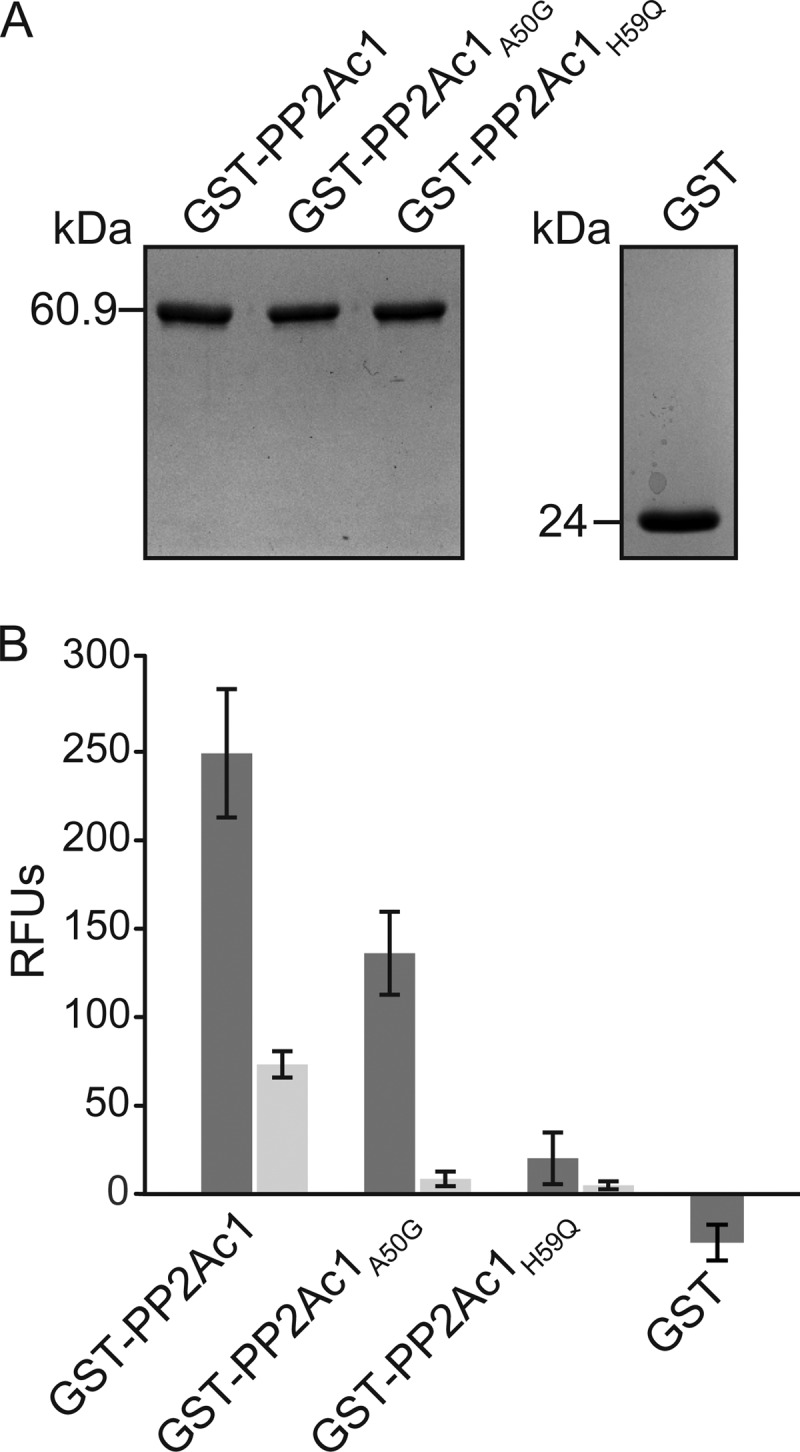
*In vitro* phosphatase activity of PP2Ac1. (A) GST-PP2Ac1, GST-PP2Ac1_A50G_, GST-PP2Ac1_H59Q_, and GST were purified from *E. coli*. (B) RFU caused by dephosphorylation of DiFMUP by PP2Ac1, PP2Ac1_A50G_, PP2Ac1_H59Q_ (*n =* 9), and GST (*n =* 6) without okadaic acid (dark gray) and with okadaic acid (light gray; *n =* 6). Error bars indicate the standard deviations of the experiments shown.

Since the catalytic subunits of PP2A are highly conserved from fungi to humans (see Fig. S1 in the supplemental material), putative residues comprising the active site of this enzyme are easily predictable. Thus, we were able to identify the active site of PP2Ac1. To study phosphatase activity, we generated a *pp2Ac1* derivative with the highly conserved histidine replaced with a glutamine at position 59 (H59Q). Mammalian homolog proteins harboring the H59Q mutation in the active site were inactive *in vitro* (29, 30). As a further control, we constructed a *pp2Ac1* derivative with a mutation at codon 50 encoding the nonconserved alanine (A50G). We purified glutathione *S*-transferase (GST)-PP2Ac1, GST-P2Ac1_A50G_, GST-PP2Ac1_H59Q_, and GST from *Escherichia coli* (Fig. 5A) and used the purified proteins in phosphatase activity tests with 6,8-difluoro-4-methylumbelliferyl phosphate (DiFMUP) as a substrate (Fig. 5B). GST-PP2Ac1 showed phosphatase activity with approximately 249 relative fluorescence units (RFU), while the activity of GST-PP2Ac1_A50G_ was slightly reduced at 136 RFU and GST-PP2Ac1_H59Q_ was impaired in phosphatase activity at only 20 RFU. GST alone did not show any phosphatase activity. To verify that the phosphatase activity detected resulted from PP2Ac1, the specific PP2A inhibitor okadaic acid was added to the reaction mixture (31). Okadaic acid reduced the phosphatase activity of GST-PP2Ac1, GST-PP2Ac1_A50G_, and GST-PP2Ac1_H59Q_ by 71, 93, and 77%, respectively (Fig. 5B). These data indicate that PP2Ac1 is an active phosphatase *in vitro* and a subunit of PP2A.

### Phosphatase activity is required for sexual development.

**FIG 6:**
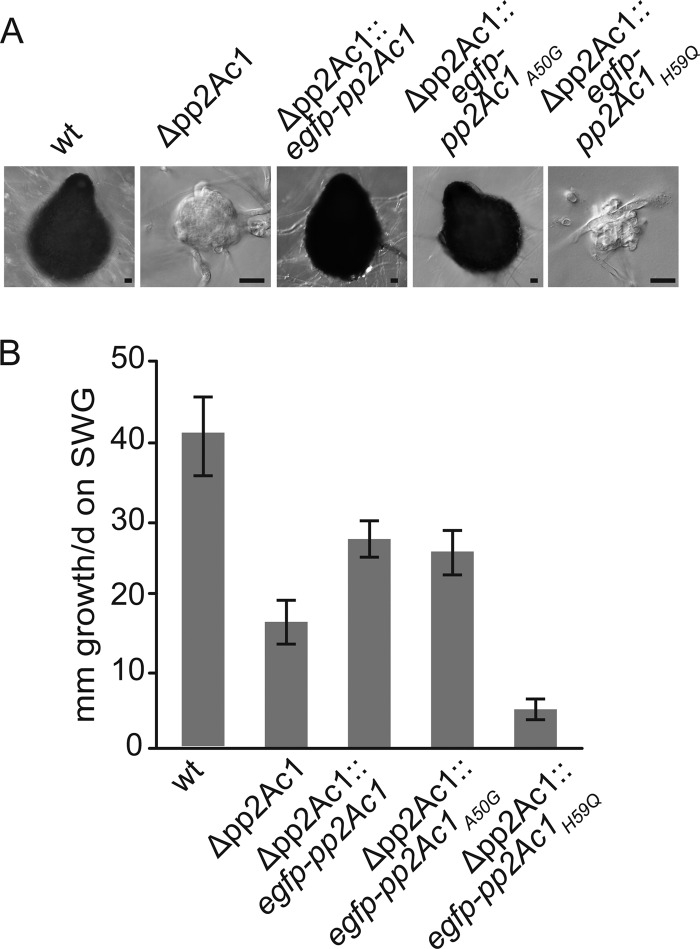
Functional *in vivo* analysis of mutated PP2Ac1. (A) DIC images of sexual development on BMM-coated slides after 7 days of growth. Scale bars, 20 µm. (B) Vegetative growth on SWG medium was measured in three independent experiments with two replicates each for 6 days in race tubes, and growth fronts were marked every 24 h. The error bars represent the standard deviations. The phenotypic analysis of the wild-type (wt) and Δ*pp2Ac1* mutant strains is depicted in Fig. 1.

The results of *in vitro* phosphatase activity tests revealed that the conserved active site of PP2Ac1 is required for enzyme activity. To study the physiological effect of modified phosphatase activity on *S. macrospora*, constructs encoding PP2Ac1, PP2Ac1_A50G_, and PP2Ac1_H59Q_ were used for comparative complementation studies with the Δ*pp2Ac1* strain as the recipient, with fully developed strains carrying the gene derivatives as indicated in Fig. 6A. While the Δ*pp2Ac1*::egfp-pp2Ac1 and Δ*pp2Ac1*::egfp-pp2Ac1_A50G_ strains formed mature wild-type-like perithecia, the Δ*pp2Ac1*::egfp-pp2Ac1_H59Q_ strain generated only protoperithecia with few enveloping hyphae. The vegetative growth defect of the *pp2Ac1* deletion strain was restored by reintroduction of the full-length wild-type gene and *pp2Ac1*_A50G_ to approximately 66% of wild-type growth. In contrast, the introduction of *pp2Ac1*_H59Q_ decreased the growth rate of the Δ*pp2Ac1* strain further by about 88% compared to that of the wild type (Fig. 6B).

## DISCUSSION

PP2Ac1, the catalytic subunit of PP2A, is a core subunit of STRIPAK, a conserved eukaryotic complex. STRIPAK regulates different aspects of eukaryotic growth. In mammalian cells, it is involved in cytoskeleton organization, cell migration, and morphology control, while in filamentous fungi it controls sexual development, vegetative growth, and hyphal fusion (14, 15). The functional analysis and enzymatic characterization of PP2Ac1 provides a more mechanistic insight into the regulation of eukaryotic developmental processes. Moreover, the data presented strongly imply that cross talk between STRIPAK and other conserved eukaryotic regulatory complexes occurs.

### The heterotrimeric phosphatase PP2A governs numerous fungal developmental processes.

The highly conserved protein phosphatase PP2A participates in a variety of cellular processes and was described as a kinase phosphatase (12). It comprises a scaffold (PP2A) and a catalytic subunit (PP2Ac) forming a core enzyme, which is joined by a regulatory subunit (PP2AB) to form a functional protein phosphatase. Either of two catalytic subunits provides PP2A phosphatase activity. By functionally characterizing the isoform PP2Ac1, we specified it as an important factor for fungal fruiting body formation, hyphal fusion, and vegetative, as well as stress-related, growth in *S. macrospora*. This agrees with findings on other filamentous fungi, where homologs of PP2Ac1 are involved in hyphal fusion, vegetative growth, and sexual and asexual development (9, 18, 21). The deletion of the *pp2Ac1* gene results in a sterile *S. macrospora* strain generating ascogonial coils and unpigmented protoperithecia. This phenotype resembles the pro phenotype described previously for other STRIPAK deletion strains and other developmental mutants (reviewed in references 14 and 22). In the ascomycetes *S. macrospora* and *Neurospora crassa*, many of these developmental mutants are impaired in hyphal fusion (22, 32). The deletion of the *N. crassa pp2Ac1* homolog led to a female sterile strain that also fails to form conidial anastomosis tubes (CATs), and shows reduced vegetative growth rates (18). CATs are specialized thin hyphae formed during conidial germination and mediate cell fusion at this developmental stage (32, 33). Reduced vegetative growth rates were also described for an *Aspergillus nidulans pp2Ac1* deletion strain that shows a defect in asexual development and is sensitive to high glucose concentrations (21). This stress-related growth defect is also found in *S. macrospora* Δ*pp2Ac1*, and we hypothesize that, similar to findings on *S. cerevisiae* and rat cell lines, large amounts of glucose activate PP2Ac1 (34, 35). Moreover, our complementation analysis with *ntap-pp2Ac1* showed that sexual development and hyphal fusion are not necessarily linked (Fig. 1A and B). Since the native construct complements all of the defects of the deletion strain, the lack of hyphal fusion might be due to overexpression of *ntap-pp2Ac1.*

Until now, evidence for the phosphatase activity of PP2Ac1 in filamentous fungi was lacking. Our *in vitro* tests with the wild-type and mutant versions of PP2Ac1 indicate clearly that the purified enzyme has enzymatic activity. We used mutant PP2Ac1_A50G_ as a positive control, since the A50G substitution affects a nonconserved residue. However, the catalytic mutant protein PP2Ac1_H59Q_ showed strongly reduced phosphatase activity, concurring with mammalian homologs sharing the same mutation (29, 30). Residue 59 (H) is highly conserved from fungi to humans and is part of the first domain of the active site (see Fig. S1 in the supplemental material). Thus, PP2Ac1 needs an intact active site for phosphatase activity. We also verified that PP2Ac1 is a catalytic subunit of PP2A, since specific inhibition by okadaic acid is concentration dependent toward PP2A, PP1, and PP2B (31, 36). The importance of phosphatase activity was demonstrated by our *in vivo* experiments in that complementation with the wild type and the control mutant version led to fertile strains with better vegetative growth than the Δ*pp2Ac1* strain, while PP2Ac1_H59Q_ was unable to restore sexual development and led to a more severe defect in vegetative growth (Fig. 5B). However, the control mutant version led to a reduced number of perithecia (see Fig. S2D in the supplemental material). In conclusion, PP2Ac1 is a phosphatase and its catalytic activity is essential for fungal sexual development and vegetative growth.

TAP-MS experiments with PP2Ac1 as bait identified additional subunits of PP2A, namely, PP2AA and CDC55. The genomes of both *N. crassa* and *S. macrospora* encode a single scaffold subunit of PP2A, PP2AA (37, 38). PP2AA is crucial for the viability of *N. crassa*, and homokaryotic ascospores of deletion mutants show apolar germination, followed by instant lysis (18). Similarly, deletion of PP2Ac2 is lethal to filamentous fungi, including *S. macrospora* (19–21). B regulatory subunit CDC55 in *A. nidulans* is involved in asexual and sexual development and septation (39), and thus, CDC55 and PP2Ac1 might form a trimeric enzyme with PP2AA to regulate septation in ascogonial coils of *S. macrospora*. The regulatory PP2A subunit found in the core STRIPAK complex is striatin, the B′′′ regulatory subunit of PP2A (14). Striatins are structurally and functionally conserved in eukaryotes and govern sexual development and pathogenicity in ascomycetes (14, 40). It has been reported that striatin homologs of animals and yeasts directly interact with PP2A catalytic subunits (15). However, we were unable to show the interaction of PP2Ac1 and the striatin homolog PRO11 by TAP-MS or Y2H analysis. Unlike in *N. crassa*, where the interaction of the PRO11 and PP2Ac1 homologs was already detected by single-step purification (18), we used a TAP-MS analysis that provides specific results but often fails to identify more-transient interactions. Furthermore, the transactivation of both pB-pp2Ac1 and pB-pro11 precludes Y2H analysis with both full-length constructs. Data from mammalian cell lines indicate that PP2AA and PP2Ac bind to the coiled-coil domains of striatins (41, 42). Together with the findings on *N. crassa*, these data indicate that binding between PP2Ac1 and PRO11 might depend on PP2AA.

### PP2Ac1 links STRIPAK to other developmental signaling pathways.

Many signaling pathways form interconnected networks that enable feedback loops and fine-tuned cross talk to control developmental processes. For STRIPAK, cross talk with the Erk pathway in insects and mammals exists (42). In *Drosophila*, the dSTRIPAK complex is important for the negative regulation of several protein kinase pathways, and STRIPAK-associated PP2A is apparently a key regulator of these processes (15).

**FIG 7:**
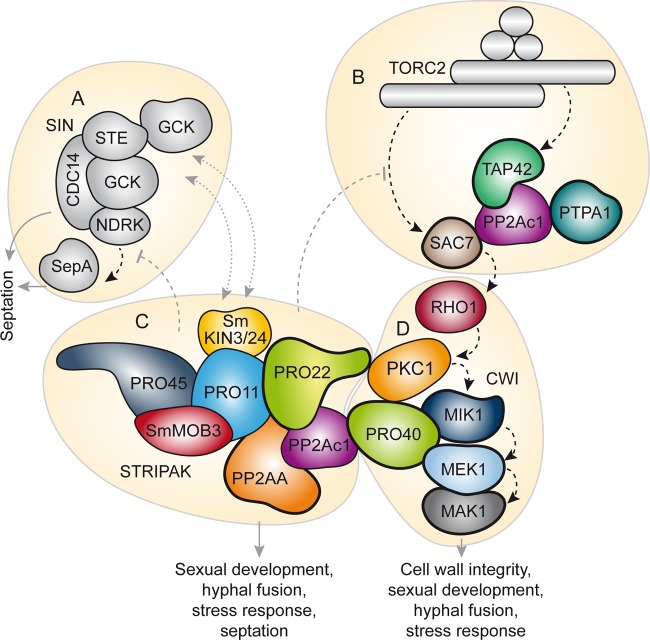
The interconnected signaling pathway of sexual development in *S. macrospora*. On the basis of functional characterization and protein-protein interaction studies, STRIPAK (C) hypothetically interacts with the SIN (A), TORC2 (B), and the CWI pathway (D). (A) The SIN is composed of three kinases and an adjacent kinase (78). Phenotypic analysis of STRIPAK mutants and TAP-MS data suggest a functional relationship between the SIN and STRIPAK in *S. macrospora* (27). (B) TORC2 targets TAP42, and SAC7 interacts with PP2Ac1. SAC7 is a GTPase-activating protein and regulates the small GTPase RHO1, a subunit of the CWI pathway (25). (C and D) Several STRIPAK and CWI pathway subunits interact, and cell wall stress-related growth defects in the Δ*pp2Ac1*, Δ*pro22*, and Δ*pp2Ac1* Δ*pro22* mutant strains point toward a relationship between the two pathways. Models of STRIPAK and CWI pathways are adapted from references 27 and 25, respectively. Interaction partners of PP2Ac1 are framed by bold lines. Solid gray arrows, dashed black arrows, dashed gray lines, and dotted gray lines indicate functions, signaling pathways, negative regulation, and putative translocation, respectively. GCK, germinal-center kinase.

Our Y2H and TAP-MS analyses indicate a connection between the STRIPAK complex and other developmental signaling pathways in fungi (Fig. 7A to D). We identified direct interactions between STRIPAK subunits PP2Ac1, PRO22, and PP2AA and subunits MIK1, MEK1, MAK1, and PRO40 of the CWI pathway (Fig. 4C and D). Our previous TAP- and AP-MS results already suggested a connection between these complexes (17, 25). The hypothesis of cross talk between STRIPAK and the CWI pathway is supported by findings on mammals and *S. cerevisiae*, where PP2A is supposedly a negative regulator of CWI pathway signaling (43, 44). Similarly, STRIPAK probably regulates MAPK signaling in filamentous fungi. In *N. crassa*, STRIPAK subunits affect the nuclear accumulation of the MAPK MAK-1 in conjunction with MAK-2, which suggests feedback loops between the STRIPAK and MAPK cascades (18). Our data, together with reports on different yeasts, further suggest that STRIPAK has strong links to the evolutionarily conserved signaling complexes target of rapamycin complex (TORC) and the septation initiation network (SIN) (Fig. 7A to C). TORC exists as a rapamycin-sensitive or -insensitive complex containing S/T protein kinase TOR1 or -2, as well as different sets of subunits (45), and regulates nutrient sensing, growth, metabolism, and aging (46, 47). In *S. cerevisiae*, where the STRIPAK-like complex is called the factor arrest (FAR) complex and controls cell cycle arrest during mating, the FAR complex negatively regulates the TORC2 signaling network (26, 48–50). In our TAP-MS analysis, we found evidence of a link between PP2Ac1 and GTPase-activating protein SAC7, a signaling component of the CWI and TORC2 pathways in *S. cerevisiae* (26, 51, 52). The physical interaction between PP2Ac1 and TAP42 is a further hint at the link between STRIPAK and TORC (Fig. 7B and C). The *S. cerevisiae* homolog of TAP42 forms a complex with PP2Ac independently of PP2AA and PP2AB and is a target of the TORC signaling pathway, and deletion of the *pp2Ac1*-like gene for PPG1 suppresses mutant alleles of TORC (50, 53, 54).

Previously published data on *S. macrospora* and *Schizosaccharomyces pombe* already underlined the connection between STRIPAK and the SIN (27, 55). Specifically, PRO45 and its *S. pombe* homolog Csc1 link the STRIPAK complex to the SIN in both fungi (Fig. 7A and C). In *S. pombe*, the STRIPAK-like complex antagonizes the SIN and was therefore called the SIN-inhibitory PP2A complex (55). In this yeast, the SIN is crucial for cytokinesis and septum formation, and asymmetry of SIN components indicates active SIN signaling during mitosis (56). The *S. macrospora* Δ*pp2Ac1*, Δ*pro22*, and Δ*pp2Ac1* Δ*pro22* deletion strains lack intercalary septa in ascogonial coils, indicating a development-specific dysregulation of the SIN in these STRIPAK mutants (Fig. 7). The structure of septa in ascogonia may vary from the septal structure in vegetative mycelium, which was shown in *Sordaria humana* (57). Intercalary septation in ascogonial coils might be essential for spatial separation or accumulation of signaling molecules in ascogenous hyphae during sexual development and may provide a link to the dikaryotic state preceding meiosis (23, 57). Septae with different molecular functions and compositions were described recently in *N. crassa*, where several septal pore-associated proteins show development-dependent localization to septal pores (58). Moreover, insights into the phosphorylation status of STRIPAK subunits will greatly increase our mechanistic understanding of phosphosignaling in eukaryotes.

In conclusion, our findings show that the catalytic activity of STRIPAK subunit PP2Ac1 is required for fungal fruiting body formation. Our interaction studies indicate that PP2Ac1 mediates cross talk between STRIPAK and other key regulatory complexes involved in nutrient signaling, CWI, and cytokinesis (Fig. 7A to D). Such information may be useful for understanding cellular development in general in higher eukaryotes.

## MATERIALS AND METHODS

### Strains and growth conditions.

*E. coli* XL1-Blue MRF′ (59), NEB5α (NEB), and TOP10 (IBA) were used for the propagation of recombinant plasmids under standard laboratory conditions (60). Recombinant proteins were expressed in *E. coli* BL21(DE3) (Stratagene) (61). Alternative plasmid construction and Y2H analysis were performed with *S. cerevisiae* PJ69-4a and PJ69-4α (62) as previously described (17, 63). The yeast strains were cultivated according to standard protocols; transgenic yeast strains were selected by prototrophy to leucine, tryptophan, or uracil; and corresponding experiments were carried out according to standard protocols (64).

The *S. macrospora* strains used in this study are listed in Table S1 in the supplemental material. Unless otherwise described, standard growth conditions, isolation of genomic DNA, and DNA-mediated transformation were performed as described previously (65, 66). The transformants were selected on nourseothricin (50 mg/ml)- and/or hygromycin B (80 U/ml)-containing medium. Growth tests in race tubes were performed for 7 days with 20 ml of SWG medium, SWG medium containing 250 µg/ml CFW, and SWG medium containing 0.6 M glucose as described before (25). Growth tests in petri dishes were carried out with 20 ml of SWG medium containing 0.6 M sorbitol, 0.6 M NaCl, 0.01% H_2_O_2_, or 100 mM dithiothreitol (DTT). For each experiment, three dishes were inoculated with an 8-mm-diameter agar plug and incubated for 2 to 3 days. The growth front was marked every 24 h (see Fig. S3C in the supplemental material).

### Generation of a *pp2Ac1* deletion strain and a *pp2Ac1pro22* double deletion strain.

To generate a Δ*pp2Ac1* strain, linearized pKO-pp2Ac1 was transformed into a Δ*ku70* strain (67). Details of plasmid construction are shown in Text S1 in the supplemental material. The primary transformants were selected for hygromycin resistance and subsequently verified by PCR (data not shown). Ascospore isolates of the Δ*pp2Ac1* strain with the wild-type genetic background were obtained by crosses against the spore color mutant fus as described elsewhere (68, 69) and verified by PCR and Southern blot analysis (see Fig. S3B and C in the supplemental material). The oligonucleotides used in this work are listed in Table S2 in the supplemental material. For a *pp2Ac1 pro22* double deletion strain, Δ*pro22* was crossed against a spore color mutant, Δ*pp2Ac1*::natp-pp2Ac1/fus. Ascospore isolates were selected for hygromycin B resistance and nourseothricin sensitivity to obtain strains with a wild-type genetic background (Fig. S3A). Strains were verified by PCR analysis (see Fig. S3B in the supplemental material).

### Production and purification of recombinant proteins.

Plasmid construction is described in Text S1 in the supplemental material. Plasmids for high expression of recombinant genes were transformed into *E. coli* BL21(DE3). *E. coli* strains were grown in LB liquid culture with ampicillin for selection while shaking at 280 rpm and 37°C until reaching an optical density at 600 nm of 0.5 to 0.6, when overexpression was induced with 100 ng/ml anhydrotetracycline for 1 h at 280 rpm and 37°C. Overexpression of the GST gene was performed with plasmid pGEX-4T-1 as previously described (70). Cells were harvested and disrupted by sonication, followed by centrifugation to clear the soluble fraction, which was subjected to SDS-PAGE and AP as described previously (70). Purification was performed with glutathione Sepharose 4 Fast Flow (GE Healthcare Life Science, Freiburg, Germany) affinity medium as previously described (70), with minor modifications during washing. The first washing step was performed in batches, and the three following washing steps were performed on a column. The elution buffer contained 30 µM glutathione and 100 mM NaCl. Elutions were concentrated, and the buffer was exchanged against 100 mM Tris/Cl, pH 8.

### TAP-MS.

For TAP, the above-mentioned Δ*pp2Ac1*::ntap-pp2Ac1 strain (A1572) was generated and analyzed for production of NTAP-PP2Ac1 by Western blotting as previously described (17). The strain was precultured in liquid BMM for 2 days and cultured for 3 days at 27°C in 12 P-flasks containing BMM, and protein extraction and TAP were performed as described previously (17). Tobacco etch virus protease cleavage was performed with AcTEV (Novex). Tryptic digestion, multidimensional protein identification technology MS, and peptide identification were performed with four replicates as described elsewhere (17).

### Y2H analysis.

The Y2H plasmids used in this study are listed in Table S3 in the supplemental material, and plasmid generation is described in Text S1 in the supplemental material. Haploid strains were generated by electroporation or chemical transformation (71, 72). PJ69-4α and PJ69-4a containing pGBKT7 and pGADT7 derivatives, respectively, were mated to generate diploid strains as previously described (73). Strains were plated on medium lacking leucine and tryptophan or leucine and uracil to analyze growth. Reporter gene activity was analyzed on medium lacking leucine, tryptophan/uracil, adenine, and histidine. Drop plating assays were performed as previously described (25). Constructs showing transactivation were omitted from the analysis; these were pB-pp2Ac1, pB-pro11, pB-Smmob3, and pB-ENT1. We tested pGBKT7 derivatives with pA-Δn-ranbpm, since the fusion protein Gal4-AD–RanBPM activates reporter gene expression with every functional Gal4 BD fusion protein (74).

### Microscopic investigations.

Microscopic investigations were performed with an AxioImager microscope (Zeiss, Jena, Germany). Sexual development and hyphal fusion were documented by differential interference contrast (DIC) microscopy with strains inoculated onto BMM-coated glass slides for 2 to 7 days or after 2 days on cellophane-covered MMS dishes, respectively (75). Fluorescence microscopy was carried out with an XBO75 xenon lamp (LEJ, Jena, Germany). For fluorescence microscopy, strains were grown on BMM-coated glass slides for 2 to 3 days (68). Fluorescence was observed with filter set 31000v2 (ET560/40x, ET630/75m, T585lp) for CFW M2R (Sigma-Aldrich).

### Phosphatase activity measurements.

The phosphatase activity of purified recombinant proteins was measured in a phosphatase activity assay with DiFMUP as the substrate (76, 77). This substrate can be dephosphorylated by a phosphatase, producing the fluorescent product 6,8-difluoro-7-hydroxy-4-methylcoumarin, showing excitation and emission maxima at approximately 358 and 450 nm (76). Thus, fluorescence intensity corresponds to the phosphatase activity of the protein tested and was measured in RFU with a fluorometer (TKO 100; Hoefer). For each sample, 1 µg of protein was used with 600 µl of buffer containing 83.3 µM difluoro-4-methylumbelliferyl phosphate (DiFMUP), 50 mM Tris (pH 8.0), 8 mM MnCl_2_, and 0.05% (wt/vol) acetylated bovine serum albumin. The reaction was performed three times in triplicate for 60 min at 30°C in the dark. Fluorescence measurements were taken after the addition of 1.9 ml of distilled water. To verify that fluorescence was due to a dephosphorylation reaction by PP2Ac1, we performed two inhibitor experiments, each in triplicate, with 5 nM okadaic acid added to the reaction mixtures or with the GST tag alone.

## SUPPLEMENTAL MATERIAL

Text S1  Supplemental materials and methods. Download Text S1, DOCX file, 0.02 MB

Dataset S1  TAP-MS with PP2Ac1 as bait. Download Dataset S1, XLSX file, 0.5 MB

Figure S1  Protein sequence alignment of *S. macrospora* (sm) PP2Ac1 and -2 (XP_003346505.1, XP_003346583.1) and *Homo sapiens* (hs) PP2AcA and -B (NP_002706.1, NP_001009552.1). The PP2A, PP4, and PP6 phosphoprotein phosphatase metallophosphatase domain (MPP_PP2A_PP4_PP6) is underlined in green. The yellow and purple boxes contain the active sites and the highly conserved C terminus, respectively. Asterisks indicate the positions of the A50G (blue) and H59Q (orange) amino acid substitutions generated in this study. %ident. is percent identity to the amino acid sequence of PP2Ac1. Download Figure S1, TIF file, 0.7 MB

Figure S2  (A) The *pp2Ac1* locus in the wild-type and Δ*pp2Ac1* mutant strains. Oligonucleotides and corresponding PCR fragments are indicated by arrows and gray lines, respectively. Restriction enzyme sites used for hydrolysis prior to Southern blot analysis are indicated, and thick gray lines show the probes used for Southern hybridization. The image is not drawn to scale. (B) Homologous integration at the 5′ flank (5′ HR) and 3′ flank (3′ HR) and the presence of *pp2Ac1* were tested in PCR assays with primer pairs KO-4678-1 and d1, KO-4678-2 and d2, and 4678-NTAP-fw and 4678-NTAP-rv, respectively. Wild-type (wt) genomic DNA served as a control, and the negative control (NC) contained no DNA. (C) Southern hybridization with radioactively labeled probes specific for *hph* and *pp2Ac1* after restriction of genomic DNA with BamHI. (D) Quantification of perithecium formation in fertile complemented Δ*pp2Ac1* mutant strains. Perithecia were counted as described in Text S1. Error bars and asterisks indicate standard deviations and *P* > 0.01, respectively. Strains not connected by brackets show significantly different numbers of perithecia (*P* < 0.01). Download Figure S2, TIF file, 1.5 MB

Figure S3  (A) The *pp2Ac1* and *pro22* loci in the wild-type and Δ*pp2Ac1* Δ*pro22* mutant strains. Arrows indicate oligonucleotides for deletion verification. Gray lines show PCR fragments. The image is not drawn to scale. (B) We verified the Δ*pp2Ac1* Δ*pro22* strain by PCR analysis with primer pairs KO-4678-1/d1, KO-4678-2/d2, 4678-NTAP-fw/4678-NTAP-rv, pro22_vp1/d1, pro22_vp2/d2, and pro22_vp2/pro22_vp3 to analyze the homologous integration of the 5′ and 3′ flanks of *pp2Ac1* and *pp2Ac1* and those of *pro22* and *pro22*, respectively. Wild-type (wt) genomic DNA served as a control, and the negative control (NC) contained no DNA. (C) Stress-related growth on SWG medium containing 100 mM DTT, 0.6 M sorbitol, 0.01% H_2_O_2_, or 0.6 M KCl in petri dishes stimulating ER, high-sugar-induced osmotic, reactive oxygen species, and high-salt-induced osmotic stress compared to growth on SWG medium. Growth tests were performed in petri dishes in triplicate, and the growth fronts were marked every 24 h for 2 to 3 consecutive days. Error bars indicate standard deviations. Download Figure S3, TIF file, 2 MB

Figure S4  Growth control for yeast strains in Y2H analyses. Shown is the growth of the strains shown in Fig. 4A (A) and 4C (B). Diploid strains were tested for growth on SD medium lacking leucine and uracil/tryptophan. nd^+^, not determined. Download Figure S4, TIF file, 1.4 MB

Figure S5  Phosphorylation of MAK1 in STRIPAK Δ*pp2Ac1*, Δ*pro22*, and Δ*pp2Ac1* Δ*pro22* mutants compared to that in the wild type (wt). Western blot analyses of MAK1 phosphorylation levels and tubulin (used as an internal standard) were done with anti-phospho-p44/42 and anti-tubulin antibodies, as depicted at the top and bottom, respectively. Download Figure S5, TIF file, 1 MB

Table S1  *S. macrospora* strains used in this study. Table S1, DOCX file, 0.02 MB

Table S2  Oligonucleotides used in this study. Table S2, DOCX file, 0.02 MB

Table S3  Plasmids used in this study. Table S3, DOCX file, 0.04 MB
