# Supplementary material for: Catalytic Subunit 1 of Protein Phosphatase 2A Is a Subunit of the STRIPAK Complex and Governs Fungal Sexual Development
Source: mBio. 2016 Jun 21;7(3):e00870-16. doi: 10.1128/mBio.00870-16 (PMC4916389; doi:10.1128/mBio.00870-16)
Supplement: Text S1 — Supplemental materials and methods. Download [file mbo003162867s1.docx]

**SUPPLEMENTARY MATERIALS AND METHODS**

**Generation of plasmids.** All oligonucleotides and plasmids used in this study are listed in Tables S2 and S3. To generate pKO-pp2Ac1, the 5’ and 3’ regions of *pp2Ac1* were amplified by PCR from genomic DNA of *S. macrospora* and with the primer pairs 4678-5-XbaI/4678-5-HindIII and 4678-3-BamHI/4678-3-NcoI, respectively. Oligonucleotides used in this work are listed in Table S2. The 5’ region and the 3’ region of *pp2Ac1* were successively inserted into the *Xba*I/*Hin*dIII and *Bam*HI/*Nco*I sites of linearized pDrive-hyg.

**Plasmid** construction for complementation and tandem affinity purification was performed by homologous recombination (hr) in yeast or ligation-mediated cloning. For pNTAP-pp2Ac1, pDS21 (12) was digested with *Xho*I/*Eco*RI and transformed in yeast together with a fragment amplified from genomic DNA using primer pair 4678-NTAP-fw/4678-NTAP-rv. Furthermore, a *pp2Ac1* fragment amplified from genomic DNA with the primers 4678-Ngfp-fw/4678-Ngfp-re was used with *Xho*I/*Eco*RI-digested pDS23 (7) for hr resulting in pEGFP-pp2Ac1. For complementation analysis with *pp2Ac1* controlled by its native promoter and terminator, a 3.6 kb fragment, harboring 1.8 kb 5’ region, the *pp2Ac1* coding region*,* and 0.8 kb 3’ region, was amplified with primers Ppp2Ac1-fw and Tpp2Ac1-rv. The fragment was digested with *Bam*HI and *Sna*BI and cloned into the *Bam*HI/*Sna*BI sites of pDrive-nat1, resulting in plasmid pNA-pp2Ac1. For the construction of plasmids encoding PP2Ac1 derivatives with the amino acid substitutions H59Q or A50G, the Q5® Site-Directed Mutagenesis Kit (NEB) was used according to the manufacturer’s instructions. For the PCR, pEGFP-pp2Ac1 was used as template with primer pairs Q5-4678-H59Q-fw/Q5-4678-H59Q-rv and Q5-4678-A50G-fw/Q5-4678-A50G-rv resulting in pQ5M1 and pQ5M2, respectively.

**For** overexpression of GST-PP2Ac1 in *E. coli*, pASG-25-pp2Ac1 was constructed with the StarGate® cloning system (IBA) according to the manufacturer’s instructions. The plasmid pA-4678 served as a PCR template with the primer pair 4678-IBA-fw/4678-IBA-rv. The mutated GST-tagged versions *pp2Ac1_H59Q_* and *pp2Ac1_A50G_* were generated analogously to the abovementioned mutated complementation vectors, resulting in pQ5M5 and pQ5M4, respectively.

**Y2H** plasmids were constructed as follows: for Y2H plasmids containing *pp2Ac1* and *tap42*, cDNA fragments were amplified with the primer pairs sm4678_eco/sm4678_bamR and 948_nde/948_eco_rv, respectively. For pA- and pB‑pp2Ac1, *pp2Ac1* was cloned into the *Eco*RI/*Bam*HI restriction sites of pGADT7 and pGBKT7, respectively. For pA- and pB‑tap42, *tap42* was inserted into the *Xho*I/*Eco*RI restriction sites of pGADT7 and pGBKT7, respectively. The plasmid pA-ptpa1 was constructed by hr. Two overlapping cDNA fragments were amplified with the primer pairs 3446_01_AD/3446_02 and 3446_04_AD/3446_03. Both fragments were transformed with *Nde*I/*Eco*RI restricted pGADT7 into the *S. cerevisiae* strain PJ69-4a. For pB-ptpa1, the plasmid pA-ptpa1 was cleaved with *Nde*I and *Eco*RI and the resulting fragment containing *ptpa1* cDNA was ligated into the *Nde*I/*Eco*RI sites of pGBKT7. The plasmids pA-ent1 and pB-ent1 were constructed analogously to the *ptpa1* plasmids using the primer pairs 3415-1-AD/3415-2 and 3415-3/3415-4-AD.

**Quantification of perithecia formation.** Strains were pre-cultured for 7 days on solid BMM. Standardized inoculums from every strain were transferred to two solid BMM plates and cultivated for further seven days at 27 °C. Four images per plate were acquired with a stereomicroscope (Stemi 2000-C; Zeiss) equipped with a digital camera (AxioCamERc 5s) and perithecia were counted in ImageJ (http://rsbweb.nih.gov/ij/).

**Analysis of MAK1 phosphorylation status.** The analysis of MAK1 phosphorylation status was performed as previously described (9). Briefly, strains were pre-cultured in liquid BMM for 2 days at 27 °C and standardized inoculums were transferred into liquid BMM and cultivated for additional three days at 27 °C and 40 rpm. Strains were harvested by filtration, powdered in liquid nitrogen, and resolved in FLAG extraction buffer with 1 % of Phosphatase-Inhibitor-Cocktails II and III (Sigma Aldrich, St. Louis, MO, USA). Cell debris were separated by centrifugation at 15000 rpm for 30 min, and equal amounts of crude protein extracts were used for SDS PAGE and Western Blotting. Phosphorylated MAK1 was detected using a polyclonal anti phospho-p44/42 antibody (Cell Signaling Technology, Inc., USA), and for an internal standard an anti-α-tubulin antibody (Sigma Aldrich, St. Louis, MO, USA, T9026) was used as described previously (9).

**REFERENCES**

1. **Pöggeler S, Kück U.** 2006. Highly efficient generation of signal transduction knockout mutants using a fungal strain deficient in the mammalian *ku70* ortholog. Gene **378:**1-10.

2. **Bloemendal S, Bernhards Y, Bartho K, Dettmann A, Voigt O, Teichert I, Seiler S, Wolters DA, Pöggeler S, Kück U.** 2012. A homolog of the human STRIPAK complex controls sexual development in fungi. Mol Microbiol **84:**310-323.

3. **Nowrousian M, Cebula P.** 2005. The gene for a lectin-like protein is transcriptionally activated during sexual development, but is not essential for fruiting body formation in the filamentous fungus *Sordaria macrospora*. BMC Microbiol **5:**64.

4. **Dirschnabel DE, Nowrousian M, Cano-Domínguez N, Aguirre J, Teichert I, Kück U.** 2014. New insights into the roles of NADPH oxidases in sexual development and ascospore germination in *Sordaria macrospora*. Genetics **196:**729-744.

5. **Louvet O, Doignon F, Crouzet M.** 1997. Stable DNA-binding yeast vector allowing high-bait expression for use in the two-hybrid system. Biotechniques **23:**816-818, 820.

6. **Kück U, Hoff B.** 2006. Application of the nourseothricin acetyltransferase gene (*nat1*) as dominant marker for the transformation of filamentous fungi. Fungal Genet Newsl **53:**9-11.

7. **Schindler D, Nowrousian M.** 2014. The polyketide synthase gene pks4 is essential for sexual development and regulates fruiting body morphology in *Sordaria macrospora*. Fungal Genet Biol **68:**48-59.

8. **Rech C.** 2007. Molekulargenetische Charakterisierung des pro22-Gens aus *Sordaria macrospora*: die funktionelle Beteiligung von PRO22 an der Zelldifferenzierung bei Ascomyceten. PhD. Ruhr-University Bochum, Germany.

9. **Teichert I, Steffens EK, Schnaß N, Fränzel B, Krisp C, Wolters DA, Kück U.** 2014. PRO40 is a scaffold protein of the cell wall integrity pathway, linking the MAP kinase module to the upstream activator protein kinase C. PLoS Genet **10:**e1004582.

10. **Tucker CL, Peteya LA, Pittman AM, Zhong J.** 2009. A genetic test for yeast two-hybrid bait competency using RanBPM. Genetics **182:**1377-1379.

11. **Kaelin WG, Jr., Krek W, Sellers WR, DeCaprio JA, Ajchenbaum F, Fuchs CS, Chittenden T, Li Y, Farnham PJ, Blanar MA, Livingston DM, Flemington EK.** 1992. Expression cloning of a cDNA encoding a retinoblastoma-binding protein with E2F-like properties. Cell **70:**351-364.

12. **Gesing S, Schindler D, Fränzel B, Wolters D, Nowrousian M.** 2012. The histone chaperone ASF1 is essential for sexual development in the filamentous fungus *Sordaria macrospora*. Mol Microbiol **84:**748-765.
