# Supplementary figures and images for: Catalytic Subunit 1 of Protein Phosphatase 2A Is a Subunit of the STRIPAK Complex and Governs Fungal Sexual Development
Source: mBio. 2016 Jun 21;7(3):e00870-16. doi: 10.1128/mBio.00870-16 (PMC4916389; doi:10.1128/mBio.00870-16)

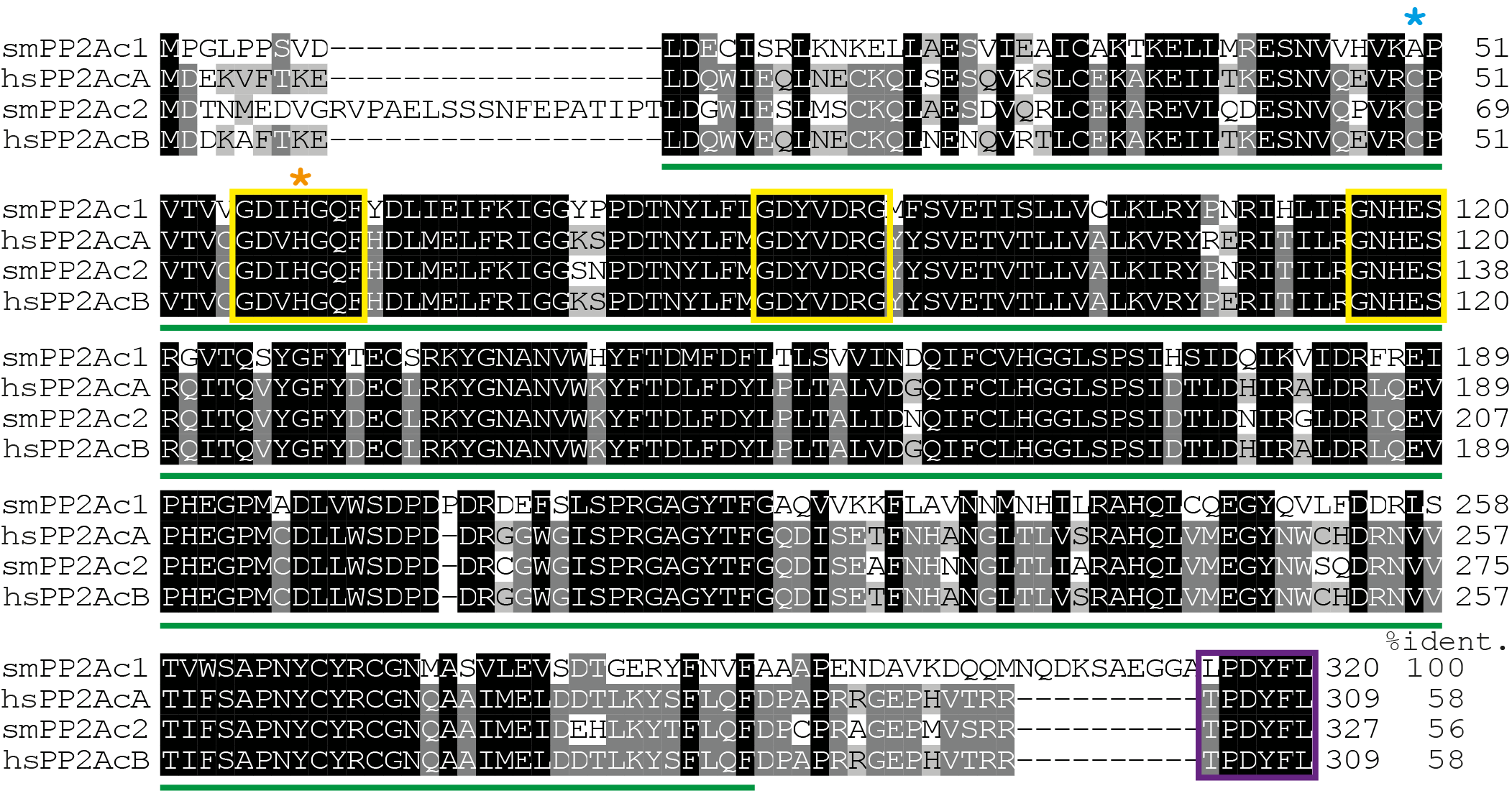

Supplement: Figure S1 — Protein sequence alignment of S. macrospora (sm) PP2Ac1 and -2 (XP_003346505.1, XP_003346583.1) and Homo sapiens (hs) PP2AcA and -B (NP_002706.1, NP_001009552.1). The PP2A, PP4, and PP6 phosphoprotein phosphatase metallophosphatase domain (MPP_PP2A_PP4_PP6) is underlined in green. The yellow and purple boxes contain the active sites and the highly conserved C terminus, respectively. Asterisks indicate the positions of the A50G (blue) and H59Q (orange) amino acid substitutions generated in this study. %ident. is percent identity to the amino acid sequence of PP2Ac1. Download [file mbo003162867sf1.tif]

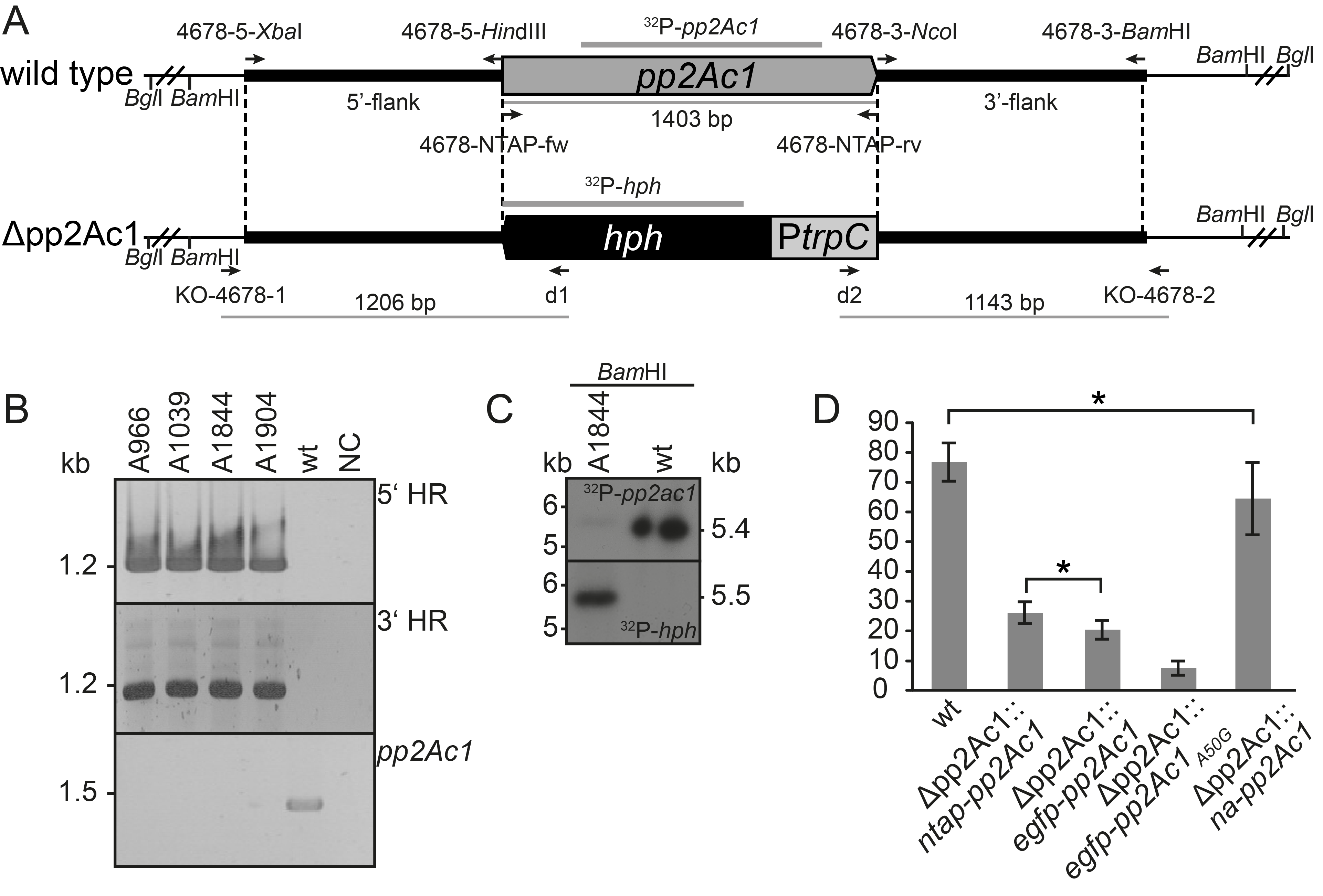

Supplement: Figure S2 — (A) The pp2Ac1 locus in the wild-type and Δpp2Ac1 mutant strains. Oligonucleotides and corresponding PCR fragments are indicated by arrows and gray lines, respectively. Restriction enzyme sites used for hydrolysis prior to Southern blot analysis are indicated, and thick gray lines show the probes used for Southern hybridization. The image is not drawn to scale. (B) Homologous integration at the 5′ flank (5′ HR) and 3′ flank (3′ HR) and the presence of pp2Ac1 were tested in PCR assays with primer pairs KO-4678-1 and d1, KO-4678-2 and d2, and 4678-NTAP-fw and 4678-NTAP-rv, respectively. Wild-type (wt) genomic DNA served as a control, and the negative control (NC) contained no DNA. (C) Southern hybridization with radioactively labeled probes specific for hph and pp2Ac1 after restriction of genomic DNA with BamHI. (D) Quantification of perithecium formation in fertile complemented Δpp2Ac1 mutant strains. Perithecia were counted as described in Text S1. Error bars and asterisks indicate standard deviations and P > 0.01, respectively. Strains not connected by brackets show significantly different numbers of perithecia (P < 0.01). Download [file mbo003162867sf2.tif]

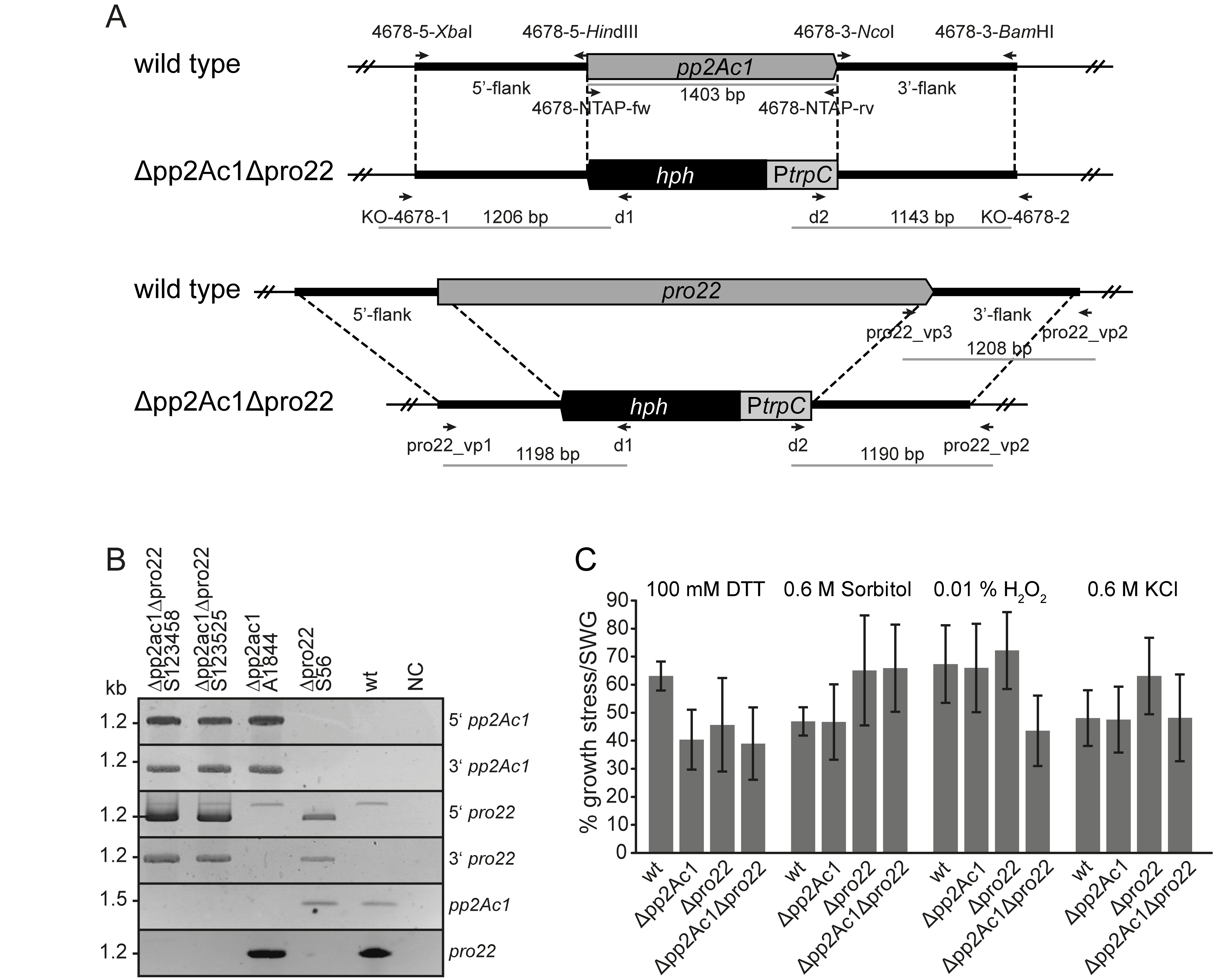

Supplement: Figure S3 — (A) The pp2Ac1 and pro22 loci in the wild-type and Δpp2Ac1 Δpro22 mutant strains. Arrows indicate oligonucleotides for deletion verification. Gray lines show PCR fragments. The image is not drawn to scale. (B) We verified the Δpp2Ac1 Δpro22 strain by PCR analysis with primer pairs KO-4678-1/d1, KO-4678-2/d2, 4678-NTAP-fw/4678-NTAP-rv, pro22_vp1/d1, pro22_vp2/d2, and pro22_vp2/pro22_vp3 to analyze the homologous integration of the 5′ and 3′ flanks of pp2Ac1 and pp2Ac1 and those of pro22 and pro22, respectively. Wild-type (wt) genomic DNA served as a control, and the negative control (NC) contained no DNA. (C) Stress-related growth on SWG medium containing 100 mM DTT, 0.6 M sorbitol, 0.01% H2O2, or 0.6 M KCl in petri dishes stimulating ER, high-sugar-induced osmotic, reactive oxygen species, and high-salt-induced osmotic stress compared to growth on SWG medium. Growth tests were performed in petri dishes in triplicate, and the growth fronts were marked every 24 h for 2 to 3 consecutive days. Error bars indicate standard deviations. Download [file mbo003162867sf3.tif]

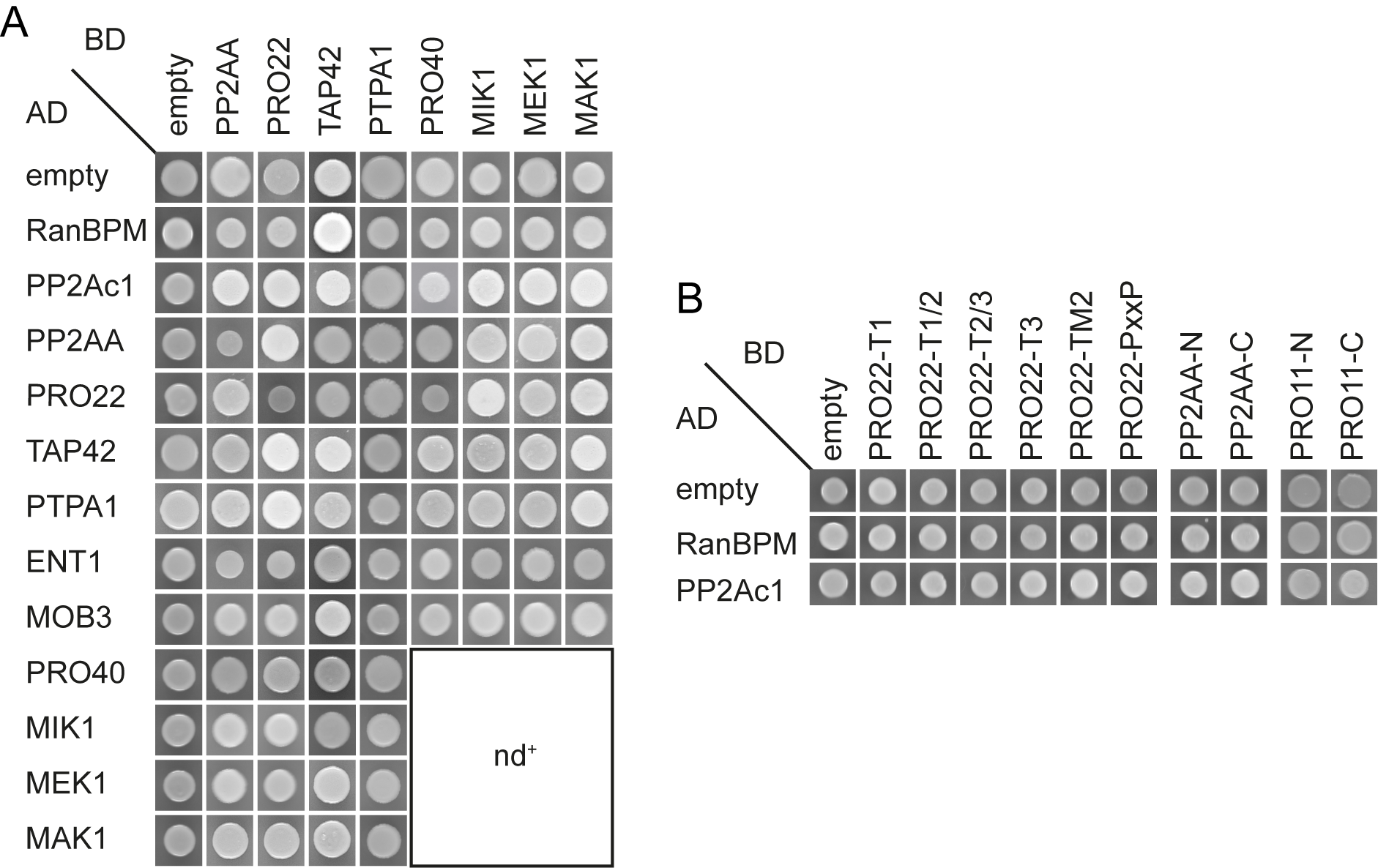

Supplement: Figure S4 — Growth control for yeast strains in Y2H analyses. Shown is the growth of the strains shown in Fig. 4A (A) and 4C (B). Diploid strains were tested for growth on SD medium lacking leucine and uracil/tryptophan. nd+, not determined. Download [file mbo003162867sf4.tif]

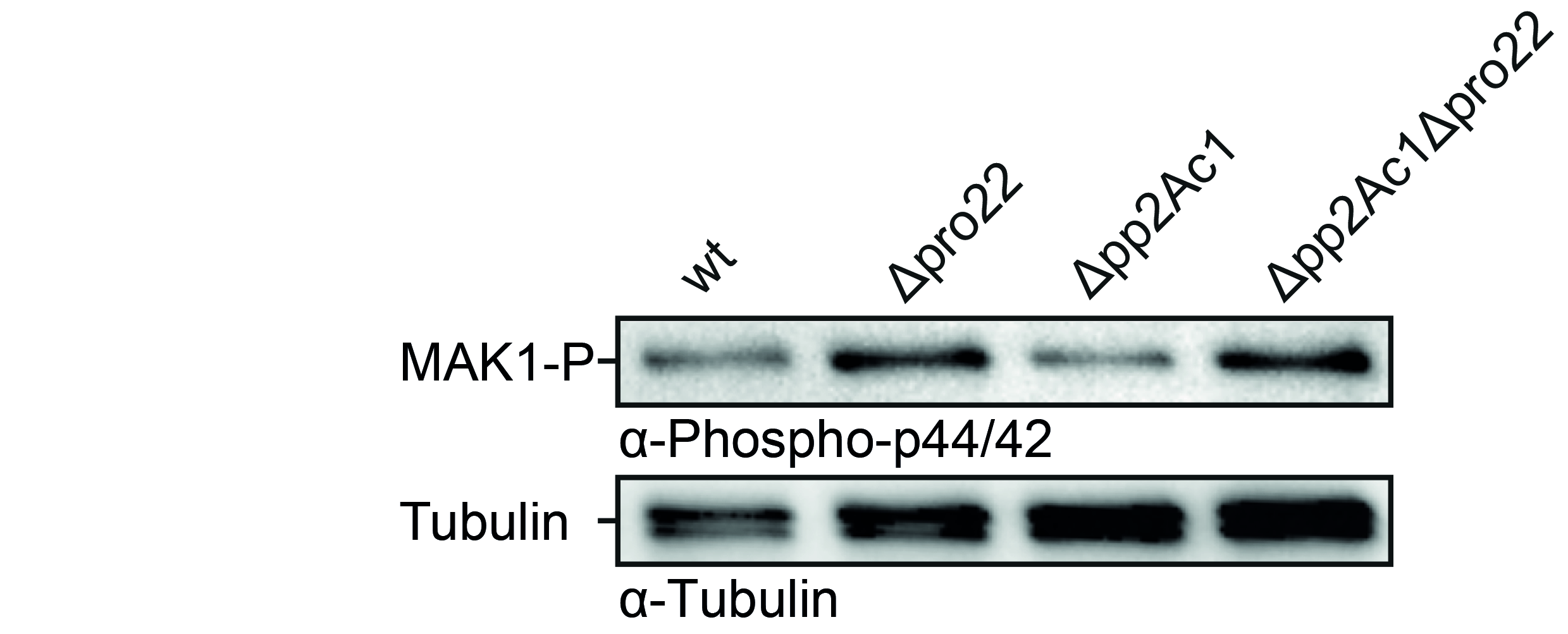

Supplement: Figure S5 — Phosphorylation of MAK1 in STRIPAK Δpp2Ac1, Δpro22, and Δpp2Ac1 Δpro22 mutants compared to that in the wild type (wt). Western blot analyses of MAK1 phosphorylation levels and tubulin (used as an internal standard) were done with anti-phospho-p44/42 and anti-tubulin antibodies, as depicted at the top and bottom, respectively. Download [file mbo003162867sf5.tif]
