# Supplementary material for: Catalytic Subunit 1 of Protein Phosphatase 2A Is a Subunit of the STRIPAK Complex and Governs Fungal Sexual Development
Source: mBio. 2016 Jun 21;7(3):e00870-16. doi: 10.1128/mBio.00870-16 (PMC4916389; doi:10.1128/mBio.00870-16)
Supplement: Table S1 — S. macrospora strains used in this study. [file mbo003162867st1.docx]

| **Strain** | **Relevant genotype and phenotype** | **Source / reference** |
| --- | --- | --- |
| S105125 | wt, fertile | culture collection^+^ |
| S96888 | Δ*ku70::nat^r^*, fertile | (1) |
| S100501 | Δ*ku70::nat^r^/fus1*, fertile | culture collection^+^ |
| S84595 | *fus1,* fertile | culture collection^+^ |
| S56 | Δ*pro22::hph^r^,* sterile | (2) |
| **A1844**, A992, A1039 | Δ*pp2Ac1::hph^r^*, sterile | This work |
| TAB72B32 | Δ*pp2Ac1::hph^r^*, *fus1,* *gpd(p)::ntap::pp2Ac1::trpc(t)::nat^r^*, fertile | This work |
| **A1572**, A1566, A1664 | Δ*pp2Ac1::hph^r^*, *gpd(p)::ntap::pp2Ac1::trpc(t)::nat^r^*, fertile | This work |
| **A1766**, A1738, A1758 | Δ*pp2Ac1::hph^r^*, *gpd(p)::egfp::pp2Ac1::trpc(t)::nat^r^*, fertile | This work |
| **A3006,** A3048, A3109 | Δ*pp2Ac1::hph^r^*, *pp2Ac1(p)::pp2Ac1::pp2Ac1(t)::nat^r^*, fertile | This work |
| A2959,  **A2966** | Δ*pp2Ac1::hph^r^*, *gpd(p)::egfp::pp2Ac1A50G::trpc(t)::nat^r^*, fertile | This work |
| **S129547**,  S130947 | Δ*pp2Ac1::hph^r^*, *gpd(p)::egfp::pp2Ac1H59Q::trpc(t)::nat^r^*, sterile | This work |
| **S123458**, S123525 | Δ*pro22::hph^r^*, Δ*pp2Ac1::hph^r^*, sterile | This work |
| S128382, T124.9 | Δ*pro22::hph^r^*, Δ*pp2Ac1::hph^r^*, *gpd(p)::pro22::mrfp1::trpc(t)::hph^r^*, *gpd::egfp::pp2Ac1::trpc(t)::nat^r^*, fertile | This work |

Bold letters indicate representative strains; *nat*^r^, nourseothricin-resistant; *hph*^r^, hygromycin resistant; ^+^culture collection of the Lehrstuhl für Allgemeine und Molekulare Botanik, Ruhr-Universität Bochum.
