# Supplementary material for: Catalytic Subunit 1 of Protein Phosphatase 2A Is a Subunit of the STRIPAK Complex and Governs Fungal Sexual Development
Source: mBio. 2016 Jun 21;7(3):e00870-16. doi: 10.1128/mBio.00870-16 (PMC4916389; doi:10.1128/mBio.00870-16)
Supplement: Table S2 — Oligonucleotides used in this study. [file mbo003162867st2.docx]

| **Oligonucleotide** | **Sequence (5‘ - 3‘)** | **Specificity** |  |
| --- | --- | --- | --- |
| sm4678_eco | TA**GAATTC**ATGCCTGGCCTTCCTCCT | cDNA *pp2Ac1* forward |  |
| sm4678_bamR | TA**GGATCC**TACAAAAAGTAATCAGGCAAAGC | cDNA *pp2Ac1* reverse |  |
| 4678-5-XbaI | **TCTAGA**GCGCCGAATCAAACCAAT | *pp2Ac1* 5‘ flank forward |  |
| 4678-5-HindIII | **AAGCTT**GGCGGGCAGAGAGA | *pp2Ac1* 5‘ flank reverse |  |
| 4678-3-NcoI | **CCATGG**TAGTCGAACAAGAGAAGG | *pp2Ac1* 3‘ flank forward |  |
| 4678-3-BamHI | **GGATCC**GCCGAGGAGGAG | *pp2Ac1* 3‘ flank reverse |  |
| 4678-NTAP-fw | GATACCGTCGACTCCATGGGGTACACTAGTATGCCTGGCCTTCCTCGTAAGTTG | *pp2Ac1* forward with overlap to pDS21 |  |
| 4678-NTAP-rv | CAGTAACGTTAAGTGGATCAGGATCCCTACAAAAAGTAATCAGGCAAAGC | *pp2Ac1* reverse with overlap to pDS21 |  |
| 4678-Ngfp-fw | TCTCGGCATGGACGAGCTGTACAAGACTAGTATGCCTGGCCTTCCTCGTAAGTTG | *pp2Ac1* forward with overlap to pDS23 |  |
| 4678-Ngfp-rv | GATTTCAGTAACGTTAAGTGGATCCCTACAAAAAGTAATCAGGCAAAGC | *pp2Ac1* reverse with overlap to pDS23 |  |
| 4678-IBA-fw | AGCGGCTCTTCAATGCCTGGCCTTCCTCCTTCGGTC | cDNA *pp2Ac1* forward for StarGate® cloning |  |
| 4678-IBA-rv_NEU | AGCGGCTCTTCTCCCCAAAAAGTAATCAGGCAAAGCCCCTC | cDNA *pp2Ac1* reverse  for StarGate® cloning |  |
| Q5-4678-H59Q-fw | TGGCGACATCcaaGGCCAGTTCTA | *pp2Ac1* mutagenesis H59Q forward |  |
| Q5-4678-H59Q-rv | ACAACGGTAACGGGGGCC | *pp2Ac1* mutagenesis H59Q reverse |  |
| Q5-4678-A50G-fw | GCATGTCAAGggcCCCGTTACCG | *pp2Ac1* mutagenesis A50G forward |  |
| Q5-4678-A50G-rv | ACAACATTACTCTCACGCATCAGCAG | *pp2Ac1* mutagenesis A50G reverse |  |
| 4678-rv_seq | AACGCTGAACATGCCTCGG | *pp2Ac1* 567-585 reverse |  |
| P4678-fw | GGATCCAGAGGACATGGCAAAGACAGG | promoter *pp2Ac1* forward |  |
| T4678-rv | CGTTACGTAGCATCTTCCGTCGTCGTCGT | terminator *pp2Ac1* reverse |  |
| 948_nde | **CATATG**AGCGACCAAGAACCTC | cDNA *tap42* forward |  |
| 948_eco_rv | **GAATTC**ACCCCCTATTCAACGTG | cDNA *tap42* reverse |  |
| 3446_01_AD | CGACGTACCAGATTACGCT**CATATG**GATACAAAATTCAAACC | cDNA *ptpa1* forward, overlap to pGADT7 |  |
| 3446_04_AD | GATGCCCACCCGGGTG**GAATTC**TTACTTGGCCCACGGCGCCT | cDNA *ptpa1* reverse, overlap to pGADT7 |  |
| 3446_02 | GATCAGGCGGCGGATGACGC | cDNA *ptpa1* reverse |  |
| 3446_03 | CGGGACACGAGTTGAGCTTCATGATG | cDNA *ptpa1* forward |  |
| 3415-1-AD | ATATGGCCATGGAGGCCAGTGAATTCATGTCCAAAGTAATTCGCAGC | cDNA *ent1* forward, overlap to pGADT7 |  |
| 3415-2 | GCTGCTGGTTCGCATATGG | cDNA *ent1* reverse |  |
| 3415-3 | GTACCAACAGCAGCAGACG | cDNA *ent1* forward |  |
| 3415-4-AD | TGCAGCTCGAGCTCGATGGATCCCGTTTAGAAAGAGATCAAATCACCC | cDNA *ent1* reverse, overlap to pGADT7 |  |
| KO-4678-1 | CAGAGAGCAACACTGAACTTCGGG | upstream of *pp2Ac1* 5‘ flank |  |
| KO-4678-2 | GGAAAAGCTGACTTGAGGTTTGTTCC | downstream of *pp2Ac1* 3‘ flank |  |
| d1 | CGATGGCTGTGTAGAAGTACTCGC | *hph*,(3) | |
| d2 | ATCCGCCTGGACGACTAAACCAA | *hph,*(4) |  |
| pro22_vp1 | CCAAGTTCAGCAACAAGAGGATGG | upstream of *pro22* 5‘ flank, (2) |  |
| pro22_vp2 | CTTACGGTAGCTACAACCCGTACA | downstream of *pro22* 3‘ flank (2) |  |
| pro22_vp3 | AATGGGAATCGGCGGAATGC | *pro22* 3505-3524 reverse (2) |  |
| 1757 | AGCTGACATCGACACCAACG | *TtrpC* reverse, Teichert pers. comm. |  |
| egfp-fw | GGTGAACTTCAAGATCCG | forward sequencing *egfp*, Teichert pers. comm. |  |
| NTAP | TTTCATAGCCGTCTCAGCAG | forward sequencing *ntap*, |  |
| Yeast1 | TCATCGGAAGAGAGTAG | forward sequencing pGBKT7 |  |
| Yeast2 | TACCACTACAATGGATG | forward sequencing pGADT7 |  |
| Yeast3 | CTTTAAAATTTGTATAC | reverse sequencing pGBKT7 |  |
| Yeast4 | GAAATTGAGATGGTGCAC | reverse sequencing pGADT7 |  |
| ASG-Primer-for | GAGTTATTTTACCACTCCCT | forward sequencing primer for pASG-IBA, IBA, Göttingen, Germany |  |
| ASG-Primer-rev | CGCAGTAGCGGTAAACG | reverse sequencing primer for pASG-IBA, IBA, Göttingen, Germany |  |
| ENTRY-Primer-for2 | GCGAAACGATCCTCGAAG | forward sequencing primer for pENTRY-IBA, IBA, Göttingen, Germany |  |
| ENTRY-Primer-rev | CCCCTGATTCTGTGGATAACCG | reverse sequencing primer for pENTRY-IBA IBA, Göttingen, Germany |  |
| SP6 | CATTTAGGTGACACTATAG | SP6 promoter forward, Eurofins, Luxembourg |  |
| T7 | TAATACGACTCACTATAGGG | T7 promoter reverse, Eurofins, Luxembourg |  |
| M13rev-49 | GAGCGGATAACAATTTCACACAGG | M13 promoter reverse, Eurofins, Luxembourg |  |

Bold and underlined letters show restriction enzyme and homologous sites, respectively.
