# Supplementary material for: Catalytic Subunit 1 of Protein Phosphatase 2A Is a Subunit of the STRIPAK Complex and Governs Fungal Sexual Development
Source: mBio. 2016 Jun 21;7(3):e00870-16. doi: 10.1128/mBio.00870-16 (PMC4916389; doi:10.1128/mBio.00870-16)
Supplement: Table S3 — Plasmids used in this study. [file mbo003162867st3.docx]

| **Plasmid** | **Relevant features** | **Reference** |
| --- | --- | --- |
| pGADT7 | Yeast two-hybrid prey plasmid, *S. cerevisiae* *PADH1* and *TADH1*, *gal4-AD* (768-881 aa), *LEU2*, *amp^r^* | Clonetech (5) |
| pGBKT7 | Yeast two-hybrid bait plasmid, *S. cerevisiae* *PADH1* and *TADH1*, *gal4-BD* (1-147 aa), *TRP1*, *kan^r^* | Clonetech (5) |
| pDrive-hyg | *trpC(p)::hph* in pDrive, *amp^r^*, *kan^r^*, *hyg^r^* | (3) |
| pD-nat1 | *trpC(p)::nat1* in pDrive, *amp^r^*, *kan^r^*, *nat^r^* | (6) |
| pDS21 | *gpd(p)::ntap::trpc(t)* in pRSnat, *URA3*, *amp^r^*, *nat^r^* | (7) |
| pDS23 | *gpd(p)::egfp::trpc(t)* in pRSnat, *URA3*, *amp^r^*, *nat^r^* | (7) |
| pKO-4678 | Deletion plasmid for *pp2Ac1*, 1 kb 5’ flank *pp2Ac1*, *trpC(p)::hph,* 0.9 kb 3’flank *pp2Ac1*, *amp^r^*, *kan^r^*, *hph^r^* | This work |
| pNTAP-4678 | *gpd(p)::ntap::pp2Ac1::trpc(t)* in pDS21, *URA3*, *amp^r^*, *nat^r^* | This work |
| pEGFP-4678 | *gpd(p)::egfp::pp2Ac1::trpc(t)* in pDS23, *URA3*, *amp^r^*, *nat^r^* | This work |
| pQ5M2 | *gpd(p)::egfp::pp2Ac1A50G::trpc(t)* in pDS23, *URA3*, *amp^r^*, *nat^r^* | This work |
| pQ5M1 | *gpd(p)::egfp::pp2Ac1H59Q::trpc(t)* in pDS23, *URA3*, *amp^r^*, *nat^r^* | This work |
| pNA-pp2Ac1 | *pp2Ac1* controlled by 1.44 kb 5’ and 0.86 kb 3’ native region in pD-nat1, *amp^r^*, *kan^r^*, *nat^r^* | This work |
| pA-4678 | *pp2Ac1* cDNA in pGADT7, *LEU2*, *amp^r^* | This work |
| pB-4678 | *pp2Ac1* cDNA in pGBKT7, *TRP1*, *kan^r^* | This work |
| pA-tap42 | *tap42* cDNA in pGADT7, *LEU2*, *amp^r^* | This work |
| pB-tap42 | *tap42* cDNA in pGBKT7, *TRP1*, *kan^r^* | This work |
| pA-ptpa1 | *ptpa1* cDNA in pGADT7, *LEU2*, *amp^r^* | This work |
| pB-ptpa1 | *ptpa1* in pGBKT7, *TRP1*, *kan^r^* | This work |
| pA-ent1 | *ent1* cDNA in pGADT7, *LEU2*, *amp^r^* | This work |
| pB-ent1 | *ent1* cDNA in pGBKT7, *TRP1*, *kan^r^* | This work |
| pA7-22FL | *pro22* cDNA in pGADT7, *LEU2*, *amp^r^* | (2) |
| pB7-22FL | *pro22* cDNA in pGBKT7, *TRP1*, *kan^r^* | (2) |
| pB-22-T1 | 1.07 kb *pro22* cDNA (aa 1-357) in  pGBDU-C1, *URA3*, *amp^r^* | (2) |
| pB-22-T1/2 | 0.77 kb *pro22* cDNA (aa 381-639) in pGBDU-C1, *URA3*, *amp^r^* | (2) |
| pB-22-T2/3 | 0.14 kb *pro22* cDNA (aa 669-715) in pGBDU-C1, *URA3*, *amp^r^* | (8) |
| pB-22-T3 | 1.14 kb *pro22* cDNA (aa 740-1120) in pGBDU-C1, *URA3*, *amp^r^* ^r^ | (2) |
| pB7-22-TM2 | 1.9 kb *pro22* cDNA (aa 1-639) in pGBKT7, *TRP1*, *kan^r^* | (2) |
| pB7-22-PxxP | *pro22* cDNA (P482A, P484-486A, P488A) in pGBKT7, *TRP1*, *kan^r^* | (2) |
| pAD-smpp2aa | *pp2aa* cDNA in pGADT7, *LEU2*, *amp^r^* | (2) |
| pBD-smpp2aa | *pp2aa* cDNA in pGBKT7, *TRP1*, *kan^r^* | (2) |
| pB7-pp2aa-N | 0.97 kb *pp2aa* cDNA (aa 1-324) in pGBKT7, *TRP1*, *kan^r^* | (2) |
| pB7-pp2aa-C | 0.93 kb *pp2aa* cDNA (aa 325-635) in pGBKT7, *TRP1*, *kan^r^* | (2) |
| pB-pro11-N | 0.84 kb *pro11* cDNA (1-281) in pGBDU-C1, *URA3*, *amp^r^* ^r^ | (2) |
| pB-pro11-C | 1.7 kb *pro11* cDNA (aa 282-845) in pGBDU-C1, *URA3*, *amp^r^* | (2) |
| pAD-smmob3 | *smmob3* cDNA in pGADT7, *LEU2*, *amp^r^* | (2) |
| pA-pro40 | *pro40* cDNA in pGADT7, *LEU2*, *amp^r^* ^r^ | (9) |
| pGBKT7-pro40 | *pro40* cDNA in pGBKT7, *TRP1*, *kan^r^* | (9) |
| pA-mik1 | *mik1* cDNA in pGADT7, *LEU2*, *amp^r^* | (9) |
| pB-mik1 | *mik1* cDNA in pGBKT7, *TRP1*, *kan^r^* | (9) |
| pA-mek1 | *mek1* cDNA in pGADT7, *LEU2*, *amp^r^* | (9) |
| pB-mek1 | *mek1* cDNA in pGBKT7, *TRP1*, *kan^r^* | (9) |
| pA-mak1 | *mak1* cDNA in pGADT7, *LEU2*, *amp^r^* | (9) |
| pA-mak1 | *mak1* cDNA in pGBKT7, *TRP1*, *kan^r^* | (9) |
| pA-Δn-ranbpm | 1.8 kb *ranbpm* cDNA (aa 51-654) in pGADT7, *LEU2*, *amp^r^* | (10) |
| pASG-25-pp2Ac1 | *gst::pp2Ac1* cDNA in pASG-IBA25, *amp^r^* | This work |
| pQ5M4 | *gst::pp2Ac1*A50G cDNA in pASG-IBA25, *amp^r^* | This work |
| pQ5M5 | *gst::pp2Ac1*H59Q cDNA in pASG-IBA25, *amp^r^* | This work |
| pGEX-4T-1 | *tac(p)::gst,* *amp^r^* | (11), GE Healthcare, Freiburg, Germany |
| pMHN2-22-1 | *gpd(p)::pro22::mrfp1::trpc(t)* in pMHN2, *amp^r^*, *hph^r^* | (8) |
